# Graphene-Reinforced PE-RT Nanocomposites for Flexible Pipeline Barriers: Structure–Property–Performance and Aging

**DOI:** 10.1021/acsomega.6c06321

**Published:** 2026-10-14

**Authors:** Lucas Galhardo Pimenta Tienne, Barbara de Salles Macena da Cruz, Vinicius de Oliveira Aguiar, Fábio Elias Jorge, Maria de Fátima Vieira Marques, Erica Gervasoni Chaves

**Affiliations:** † Institute of Macromolecules Professor Eloisa Mano (IMA), Federal University of Rio de Janeiro, Av. Horácio Macedo 2090, Rio de Janeiro, Rio de Janeiro CEP 21941-598, Brazil; ‡ Leopoldo Americo Miguêz de Mello Research and Development Center, PETROBRAS/CENPES, Av. Horácio Macedo 950, Rio de Janeiro, Rio de Janeiro CEP 21941-915, Brazil

## Abstract

Polyethylene of raised temperature resistance (PE-RT Type II) is the preferred material for barrier layers in offshore flexible risers, yet its CO_2_ permeability and long-term stability under hydrocarbon exposure remain critical performance gaps. Here, PE-RT nanocomposites reinforced with four graphene-based fillers—graphene oxide (GO), reduced graphene oxide (rGO), vinyltrimethoxysilane-functionalized GO (GOS), and graphene nanoplatelets (GNP)—at 0.5–2.0 wt % were prepared by melt compounding and systematically characterized. Filler surface chemistry and structure were characterized by Fourier transform infrared spectroscopy (FTIR), X-ray diffraction (XRD), Raman spectroscopy, thermogravimetric analysis (TGA), and scanning electron microscopy coupled with energy-dispersive X-ray spectroscopy (SEM/EDS), revealing distinct structural and interfacial features among the graphene-based fillers. SEM provided qualitative information on filler morphology and microscale distribution, while Raman spectroscopy and XRD were used to characterize the structural features of the graphene-based fillers. Because nanoscale dispersion within the PE-RT matrix was not directly quantified by TEM, AFM, or other image-based methods, relationships between filler dispersion and functional performance are discussed as structure–property interpretations rather than direct measurements of nanoscale dispersion. GOS delivered the greatest CO_2_ barrier improvement, reducing the mean permeability by 56.5% at 1 wt % relative to neat PE-RT (2.13 ± 0.30 vs 4.90 ± 0.10 cm^2^ s^–1^ psi^–1^; Tukey HSD, *p* < 0.001). The Nielsen tortuosity model yielded an effective platelet aspect ratio of ≈570, consistent with efficient platelet-induced tortuosity. GNP exhibited the highest mean stiffness at 1 wt %, with an elastic modulus increase of approximately 50.8% relative to neat PE-RT. Six months of immersion aging in synthetic hydrocarbon oil (ISO 23936-1, 80 °C) produced formulation-dependent changes in thermal, surface, dimensional, and molar-mass-related properties. The graphene-containing nanocomposites showed lower swelling than neat PE-RT, while GOS exhibited comparatively stable GPC parameters over the aging period. These results demonstrate enhanced aging resistance, although the specific mechanisms responsible for this stabilization were not directly determined. GNP showed comparatively poorer CO_2_ barrier performance despite exhibiting the highest mean mechanical properties among the 1 wt % nanocomposites. These results establish quantitative structure–property–performance relationships and provide design guidelines for graphene-functionalized polyolefin barrier materials in demanding service environments.

## Graphical abstract

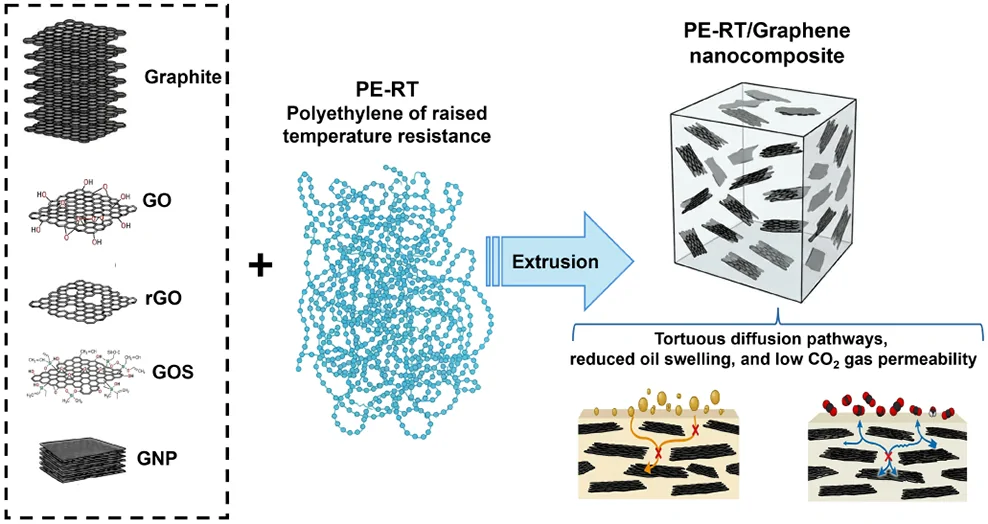

## 1 Introduction

Flexible risers in deepwater oil and gas production are multilayer composite structures in which the polymeric barrier layer must simultaneously prevent CO_2_ permeation and withstand prolonged exposure to hydrocarbon condensates at high temperatures. Polyethylene of increased temperature resistance (PE-RT) has progressively displaced high-density polyethylene (PE100) in this application owing to its bimodal molar mass distribution, higher tie-molecule density, and superior resistance to slow crack growth under sustained pressure.^1–3^ Unlike conventional HDPE, PE-RT Type II presents a less ordered crystalline morphology that responds differently to nanofiller incorporation; findings from HDPE-based systems therefore cannot be directly extrapolated to PE-RT.^3^ Despite these structural advantages, PE-RT still exhibits CO_2_ permeability and long-term thermo-oxidative stability that fall short of increasingly demanding subsea service specifications, motivating the search for effective reinforcement strategies.

Incorporation of high-aspect-ratio, two-dimensional nanofillers is a well-established route to simultaneously improving gas barrier performance and mechanical properties in semicrystalline polymers.^4,5^ Among the available options, graphene-based materials are particularly attractive: their exceptional in-plane stiffness (≈1 TPa), impermeability to gases, and large specific surface area can, when combined with adequate dispersion, create highly tortuous diffusion pathways for permeating molecules and reinforce the polymer matrix at low loadings.^4,5^ The family of graphene derivatives—graphene oxide (GO), reduced graphene oxide (rGO), and surface-functionalized variants such as silanized GO (GOS)—extends this potential by offering tunable surface chemistry that can be tailored to specific matrix polarities and processing conditions.^6,7^

PE-RT, however, presents a particular interfacial challenge: its nonpolar backbone offers few sites for adhesion to GO’s oxygenated surface, and conventional melt-compounding conditions can promote nanofiller restacking and agglomeration, negating the high-aspect-ratio advantage.^8^ Surface functionalization is therefore not merely beneficial but essential. Vinyltrimethoxysilane (VTMS) grafting onto GO (producing GOS) offers a dual mechanism: silane hydrolysis generates reactive silanol groups capable of bridging the inorganic filler surface and the polymer interface, while the vinyl moiety provides an organic segment compatible with the polyolefin matrix.^9^ Despite this mechanistic rationale, quantitative experimental evidence of GOS performance in PE-RT, particularly under simultaneous mechanical, barrier, and aging evaluation, remains scarce in the open literature.

Previous studies have demonstrated that the performance of graphene-based polymer nanocomposites is strongly governed by filler surface chemistry, dispersion, and interfacial compatibility. Nisar et al. investigated graphene-based nanostructures in polypropylene-based nanocomposites and demonstrated that filler characteristics and incorporation influence the thermal, crystallization, and mechanical behavior of the resulting materials.^10^ Similarly, Nisar et al. demonstrated that rGO-based hybrid nanostructures can be effectively incorporated into polyethylene^11^ and, more recently, into poly­(lactide),^12^ producing multifunctional nanocomposites with modified mechanical, thermal, electrical, and/or magnetic properties. Surface functionalization provides an additional route to improve filler–matrix interactions. Cruz et al., for example, functionalized GO with alkylamines of different chain lengths and demonstrated that tailoring the GO surface improved its compatibility with poly­(lactide), resulting in enhanced mechanical, thermal, and barrier properties.^11^ These studies collectively highlight that the chemical state and surface modification of graphene derivatives are critical parameters controlling their dispersion and reinforcing efficiency in polymer matrices.^10–13^ Recent studies have further emphasized the importance of linking graphene structure and surface chemistry to composite performance using complementary structural, spectroscopic, morphological, thermal, and mechanical characterization.^14–17^ Although these studies involve matrices other than PE-RT, they collectively demonstrate that graphene oxidation state, surface chemistry, and concentration can strongly influence filler–matrix interactions and the resulting material properties. However, these findings cannot be directly extrapolated to PE-RT barrier layers, for which the combined effects of graphene surface chemistry on CO_2_ transport, mechanical reinforcement, and hydrocarbon-aging behavior remain insufficiently established.

For polyolefin barrier applications, Medeiros et al. reported that incorporating low graphene contents (0.05 to 0.3 wt %) into LDPE by melt extrusion increased crystallinity and improved oxygen barrier performance by approximately 26%, illustrating the effectiveness of two-dimensional carbon fillers in increasing gas-transport tortuosity.^18^ More recently, graphene nanoplatelets incorporated into HDPE have also been shown to improve gas barrier and mechanical performance, with filler dispersion, interfacial interactions, surface area, and loading playing key roles in determining the magnitude of these effects.^19,20^

Despite these advances, the influence of graphene surface chemistry on the combined barrier, mechanical, and aging performance of PE-RT remains insufficiently understood. Previous graphene/polyolefin studies have predominantly focused on PP, LDPE, or HDPE and have generally examined individual graphene derivatives or specific property classes under unaged conditions.^10–20^ The specific research question addressed here is whether modification of graphene surface chemistry can improve the overall performance of PE-RT and how the resulting responses differ among oxidized, reduced, silane-functionalized, and commercial graphitic fillers. To address this question, GO, rGO, VTMS-functionalized GO (GOS), and commercial GNPs were incorporated into the same PE-RT Type II matrix under identical melt-processing conditions and evaluated within a common experimental framework. The novelty, therefore, lies not in graphene reinforcement of polyethylene per se, but in the direct comparison of these distinct graphene surface-chemistry states and in relating them to CO_2_ transport, mechanical and thermal properties, and stability during 6 months of hydrocarbon aging at 80 °C under ISO 23936-1 conditions. The results demonstrate that graphene incorporation alone does not ensure improved barrier performance: GOS produced a pronounced reduction in CO_2_ permeability, consistent with the combined effects of its modified surface chemistry and effective platelet-induced tortuosity, whereas the other graphene derivatives exhibited markedly different transport responses. This integrated comparison enables identification of the formulation providing the best balance of barrier, mechanical, and aging performance for PE-RT flexible-riser barrier applications.

## 2 Experimental Section

### 2.1 Materials

Commercial graphite powder (Graflake 99550, Nacional de Grafite Ltda., Brazil; particle size ≈300 μm, carbon content ≈99.8%) was used as the precursor for filler synthesis. Sodium nitrite (≥98%), sulfuric acid (95–98%), nitric acid (65%), potassium permanganate (≥99%), hydrogen peroxide (30% w/w), and hydrochloric acid (37%) were purchased from Proquimios Com. e Ind. (Brazil). Vinyltrimethoxysilane (VTMS, ≥98%) was obtained from Sigma-Aldrich. Graphene nanoplatelets (GNP) were provided by MG Grafeno (Brazil) and consisted of 95.1% graphene and 3.6% nonionic surfactant, with a typical layer distribution of 6–10 stacked sheets. The polymer matrix was Intrepid 2499 (Dow Chemical Company, USA), a bimodal PE-RT Type II produced via the UNIPOL II gas-phase process, hereafter referred to as PE-RT. For aging tests, a synthetic oil was formulated by mixing 70% heptane (≥99%), 20% cyclohexane (≥99%), and 10% toluene (≥99.5%) (v/v), following ISO 23936-1.^21^

### 2.2 Synthesis of Graphene-Based Fillers

Graphene oxide (GO) was synthesized from Graflake graphite following a procedure adapted from Hou et al.^22^ Briefly, 5.0 g of graphite flakes was transferred to a 1 L Erlenmeyer flask and mixed with an acidic solution of concentrated sulfuric acid (H_2_SO_4_, 95–98%) and nitric acid (HNO_3_, 65%) at a 3:1 volume ratio. The mixture was maintained under magnetic stirring in an ice bath for 3 h to promote graphite intercalation. Subsequently, 4.5 g of sodium nitrite (NaNO_2_, ≥98%) was added, and the suspension was maintained under magnetic stirring in the ice bath for an additional 3 h. Then, 22.5 g of potassium permanganate (KMnO_4_, ≥99%) was slowly added over a period of 2 h, while maintaining the reaction mixture under controlled conditions. After completion of the KMnO_4_ addition, the ice bath was removed, and the reaction mixture was continuously stirred for 7 days at room temperature. The resulting oxidized material was washed with distilled water by repeated centrifugation (4000 rpm, 15 min per cycle) until the pH was approximately 7. The recovered GO was subsequently dried in an oven at 80 °C for 24 h.

Reduced graphene oxide (rGO) was obtained by thermal reduction of previously prepared GO. Dried GO was placed in a furnace and subjected to thermal treatment at 1000 °C for 3 min under an N_2_ atmosphere. The thermal treatment promoted rapid expansion and reduction of GO, yielding a dark powder. After the thermal treatment, the material was cooled in a desiccator and stored until use. These conditions were selected to promote rapid thermal deoxygenation and expansion of GO, as high-temperature thermal treatment is commonly employed to obtain reduced graphene oxide.

Vinyltrimethoxysilane-functionalized graphene oxide (GOS) was prepared by a silanization procedure adapted from Tommasini et al.^23^ Briefly, 1 g of GO was dispersed in 20 mL of toluene in a 50 mL flask. The dispersion was subjected to 30 min of sonication prior to silane addition. Subsequently, 1.07 mL of vinyltrimethoxysilane (VTMS, ≥98%) was added to the GO dispersion. The reaction mixture was heated to 90 °C and maintained under constant stirring for 5 h under N_2_ atmosphere. After completion of the reaction, the functionalized graphene oxide was recovered by filtration and washed with toluene and ethanol to remove residual unreacted species. The resulting GOS was dried in a vacuum oven at 40 °C for 2 h and stored until use.

Commercial graphene nanoplatelets (GNPs) were used as received without further chemical modification.

### 2.3 Preparation of PE-RT Nanocomposites

Nanocomposites were melt-compounded using a Haake MiniLab II counter-rotating twin-screw extruder (180 °C, 100 rpm, 6-min residence time). The conical intermeshing geometry promotes elongational flow components capable of partially disrupting nanofiller agglomerates. The neat PE-RT matrix was processed under identical conditions to serve as a reference. Filler loadings of 0.5, 1.0, and 2.0 wt % were selected to cover a low-to-moderate concentration range in which graphene-based fillers can modify polymer properties while limiting restacking and agglomeration typically associated with higher nanofiller contents. This range also enabled evaluation of possible nonmonotonic loading effects, with 1.0 wt % representing the intermediate composition for direct comparison among the four filler types. Concentrations below 0.5 wt % and above 2.0 wt % were not systematically investigated in the present study.

Formulations are designated as I­[filler]­[loading]: e.g., IGOS1.0 for 1.0 wt % GOS in PE-RT.

### 2.4 Aging Protocol

Aging tests followed ISO 23936-1:2022 ^21^ using 1.0 wt % nanocomposites of GO, rGO, and GOS, which were immersed in synthetic oil at 80 °C inside sealed 2-L steel reactors for up to 6 months. The aging study was restricted to the 1.0 wt % formulations because this intermediate loading provided a common basis for comparing the effects of filler surface chemistry while avoiding the lower filler content at 0.5 wt % and the greater tendency toward filler restacking and agglomeration at 2.0 wt %. At predetermined intervals, specimens were removed, lightly surface-dried, and allowed to rest for 20 min prior to mass recording. The swelling degree (α) was calculated from the relative mass increase.^24^

### 2.5 Characterization

Thermogravimetric analysis (TGA) was performed using a TA Instruments Q500 thermogravimetric analyzer (TA Instruments, New Castle, DE, USA) under N_2_ at a heating rate of 10 °C min^–1^ up to 700 °C. X-ray diffraction (XRD) measurements were carried out using a Rigaku MiniFlex diffractometer (Rigaku Corporation, Tokyo, Japan) with Cu Kα radiation (λ = 1.5458 Å), over a 2θ range of 2–60°. Crystallite size was estimated using the Scherrer equation, and interplanar spacing was calculated using Bragg’s law. Fourier-transform infrared spectroscopy (FTIR) spectra of the graphene-based fillers were recorded from KBr pellets over the range 4000–600 cm^–1^ at a resolution of 4 cm^–1^. Polymer-film samples were analyzed in ATR mode over the same spectral range, using a resolution of 2 cm^–1^. Raman spectra were obtained using a confocal Raman spectrometer/microscope equipped with a 532 nm laser operated at 10 mW, a 20× objective, and an N_2_-cooled CCD detector. Spectra were collected over 70–3710 cm^–1^ with a spectral resolution of 3–5 cm^–1^, using 10 coadditions and an integration time of 10 s. Scanning electron microscopy coupled with energy-dispersive X-ray spectroscopy (SEM/EDS) was performed using a Quanta 200 scanning electron microscope (SEI, CETEM, Brazil), operated at 15 kV. Powder samples were mounted on carbon tape and excess powder was removed using an N_2_ stream. Composite samples were cryofractured after immersion in liquid N_2_ for 1 h, and the fractured surfaces were carbon-coated under ultrapure Ar for 150 s at 30 mA before analysis.

Composite characterization: Thermogravimetric analysis (TGA) was performed using a Q500 thermogravimetric analyzer (TA Instruments, USA) with 4–6 mg ribbon samples, from room temperature to 700 °C at 10 °C min^–1^ under N_2_ at a flow rate of 60 mL min^–1^. Differential scanning calorimetry (DSC) was carried out using a Q1000 calorimeter (TA Instruments, USA) with 4–6 mg samples sealed in aluminum pans. Samples were subjected to a heating–cooling–heating cycle between 25 and 250 °C at 10 °C min^–1^ under N_2_. The first heating scan was used to erase the previous thermal history; crystallization temperature (*T*_c_) was determined from the cooling scan, whereas melting temperature (*T*_m_) and melting enthalpy (Δ*H*_m_) were obtained from the second heating scan. The degree of crystallinity (*X*_c_) was calculated as Δ*H*_m_/(Δ*H*_m_° × *W*_p_), using Δ*H*_m_° = 293 J g^–1^ for 100% crystalline polyethylene.^25^ XRD measurements were performed on compression-molded films prepared at 180 °C for 6 min under the same instrumental conditions described above.

SEM was used for a qualitative assessment of filler morphology and microscale distribution. Because the spatial resolution and contrast of the present SEM analysis do not permit a quantitative assessment of individual graphene sheets within the PE-RT matrix, nanoscale dispersion and exfoliation were not directly quantified.

Water contact angle measurements were carried out on compression-molded films using an OCA 15 PLUS optical contact angle measuring system (DataPhysics Instruments GmbH, Filderstadt, Germany). Two deionized water droplets (∼5 μL each) were analyzed per sample, with two contact angle measurements obtained per droplet, yielding four measurements per sample. The droplet images were captured by a camera, and the contact angles were calculated automatically using the connected analysis system. The films were prepared by compression molding at 180 °C for 6 min under a pressure of 6.9 × 10^7^ Pa and subsequently cooled to 30 °C. Shore D hardness was measured according to ASTM D2240 ^26^ using a Shore D tester from Shore Instruments & MFG (Japan); five measurements were performed for each sample. Tensile testing was performed according to ASTM D638 ^27^ using Type V specimens on an Emic L-3000 universal testing machine (Emic, Brazil) equipped with a 30 kN load cell, at a crosshead speed of 10 mm min^–1^; 12 specimens were tested for each composition.

CO_2_ permeability was measured using a custom-built high-pressure permeation system equipped with a stainless-steel permeation cell, a pressure-control valve, a temperature-controlled chamber, a pressure sensor, pressure-release valves, and a data-acquisition system. Compression-molded disk specimens with a radius of approximately 2 mm, a thickness of approximately 2 mm, and an exposed area of approximately 0.126 cm^2^ were used. Prior to testing, the specimens were conditioned at 40 °C for 24 h and subsequently transferred to a desiccator and allowed to equilibrate to room temperature. Permeability measurements were performed at 90 °C under an inlet CO_2_ pressure of 50 bar for approximately 24 h. The downstream pressure was continuously recorded as a function of time. Steady-state permeation was identified from the linear region of the downstream pressure-versus-time curve following the initial transient regime. The diffusion coefficient was determined using the time-lag method, and the permeability coefficient was calculated from the steady-state pressure increase rate in the permeate compartment. The corresponding equations and calculation procedure are provided as Equations S1–S3 in the Supporting Information.

High-temperature gel permeation chromatography (HT-GPC) was used to determine the number-average molar mass (*M*_n_), weight-average molar mass (*M*_v_), and dispersity (*Đ*) of the PE-RT nanocomposites before and during aging. The high-temperature analytical conditions were selected to ensure complete dissolution of the polyethylene-based samples prior to analysis. No separation into soluble and insoluble fractions was applied. The analysis was carried out using an Agilent 1260 Infinity II high-temperature GPC system equipped with a refractive index detector and an Agilent PLgel 10 μm Mixed-B column (300 × 7.5 mm, PN PL1110–6100). Measurements were performed in duplicate at 140 °C using 1,2,4-trichlorobenzene as the eluent at a flow rate of 1.0 mL min^–1^, with polystyrene standards for calibration.

Statistical analysis. Quantitative results obtained from replicated measurements are reported as mean ± standard deviation. Tensile properties were determined from 12 independent specimens per formulation (n = 12), whereas CO_2_ permeability measurements were performed using three independent specimens per formulation (n = 3). Contact-angle characterization comprised four measurements per sample, and Shore D hardness was based on five measurements per formulation. Statistical differences among neat PE-RT and the GO-, rGO-, GOS-, and GNP-containing nanocomposites were evaluated by one-way analysis of variance (ANOVA), followed by Tukey’s honestly significant difference (HSD) post hoc test for data sets with sufficient independent replication. Differences were considered statistically significant at *p* < 0.05. DSC and TGA data, for which independent replicate measurements were not available in sufficient number for inferential statistical analysis, were evaluated descriptively.

## 3 Results and Discussion

### 3.1 Structural Characterization of Graphene-Based Fillers

The four fillers exhibit distinct thermal, structural, spectroscopic, and morphological characteristics that may influence their behavior in the PE-RT matrix.

#### 3.1.1 TGA

Graphite is thermally stable to ≈700 °C. GO shows the characteristic three-step degradation: moisture evaporation below 125 °C, decomposition of hydroxyl, epoxy, and carboxyl groups between 125–300 °C, and removal of residual oxygen functionalities above 300 °C, in agreement with Alkhouzaam et al.^28^ and Cobos et al.^29^ GOS exhibits a thermal decomposition profile similar to that of GO, with slight shifts in the DTG features. These changes alone cannot be considered direct evidence of siloxane grafting; however, when combined with the FTIR results and the detection of 2.98 wt % Si by EDS, they support the successful incorporation of VTMS-derived species onto the GO surface.^30^ rGO exhibits significantly improved thermal stability with minimal mass loss below 600 °C, confirming efficient deoxygenation during thermal reduction.^31^ GNP shows two events, moisture desorption near 100 °C and residual oxygen species near 200 °C, with overall higher stability reflecting its low oxidation degree^32^ (Figure 1).

**Figure 1:**
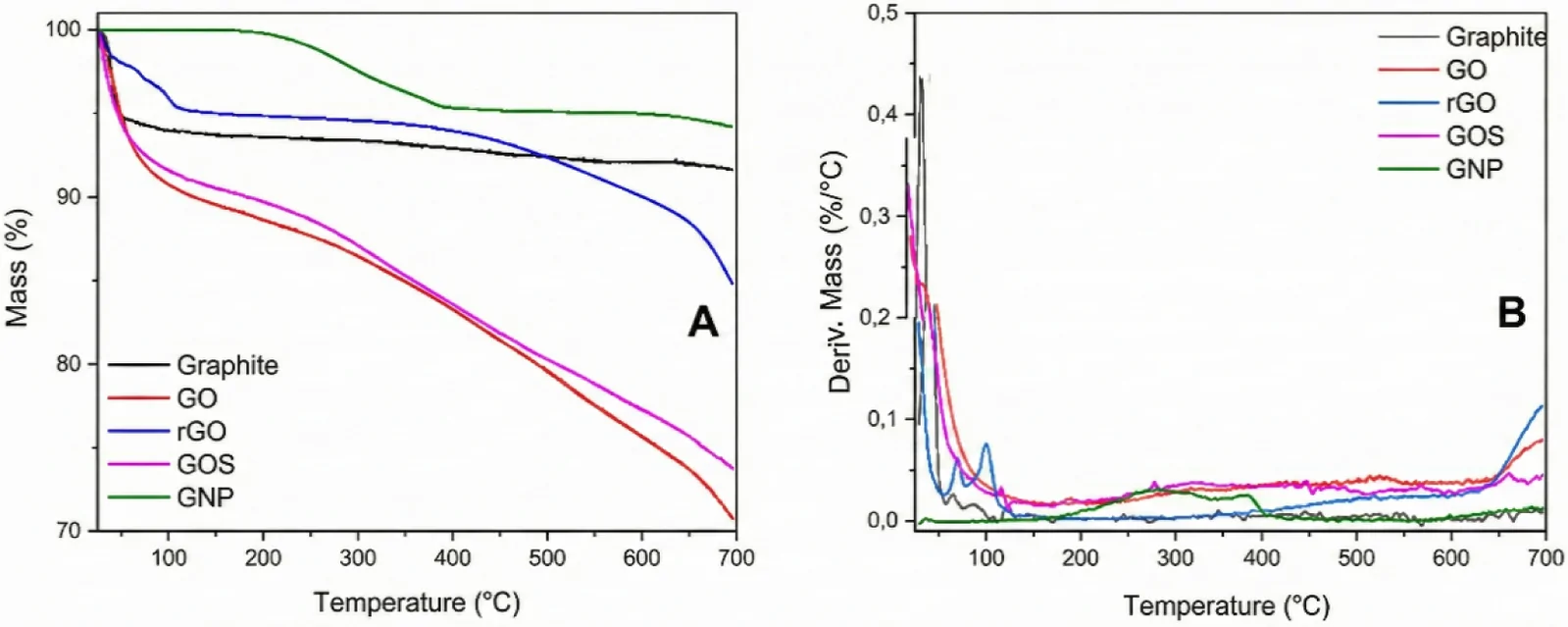
Thermal analysis of graphite and graphene-based fillers: (a) TGA curves and (b) DTG curves of graphite, GO, rGO, GOS, and GNP.

#### 3.1.2 XRD

Pristine graphite exhibits a sharp and intense (002) reflection at 2θ ≈ 26.43° (*d* ≈ 0.34 nm, *L*_c_ = 22.6 nm), characteristic of the highly ordered stacking of graphitic layers.^33,34^ In contrast, the GO pattern shows a markedly broadened and less intense diffraction feature centered at approximately 26.35° (*L*_c_ = 6.2 nm), indicating substantial disruption of the long-range graphitic order during oxidation. Notably, the characteristic low-angle (001) reflection commonly reported for GO at approximately 9°–12° is not clearly observed in the present sample. Therefore, the feature at 26.35° is not assigned to the characteristic GO structure but rather to residual graphitic domains and/or incompletely oxidized stacked regions that retain an interlayer spacing close to that of graphite.^35,36^ The rGO sample exhibits a broad reflection centered at approximately 26.59° (*L*_c_ = 5.9 nm), consistent with partial restoration of graphitic stacking following thermal deoxygenation, although the small crystallite size indicates that substantial structural disorder remains.^31^ GOS also displays a broad diffraction feature near 26.07° (*L*_c_ = 7.5 nm). The slight shift and broadening relative to graphite indicate disruption of the graphitic stacking; however, these XRD changes alone are insufficient to demonstrate covalent attachment of VTMS. Evidence for silanization is instead provided by the combined FTIR and EDS results, including the detection of 2.98 wt % Si in GOS. In contrast, GNP exhibits a sharp and intense (002) reflection at 26.65° (*L*_c_ = 19.1 nm), consistent with a substantially higher degree of graphitic stacking order than that observed for GO, rGO, and GOS (Figure 2, Table S1).

**Figure 2:**
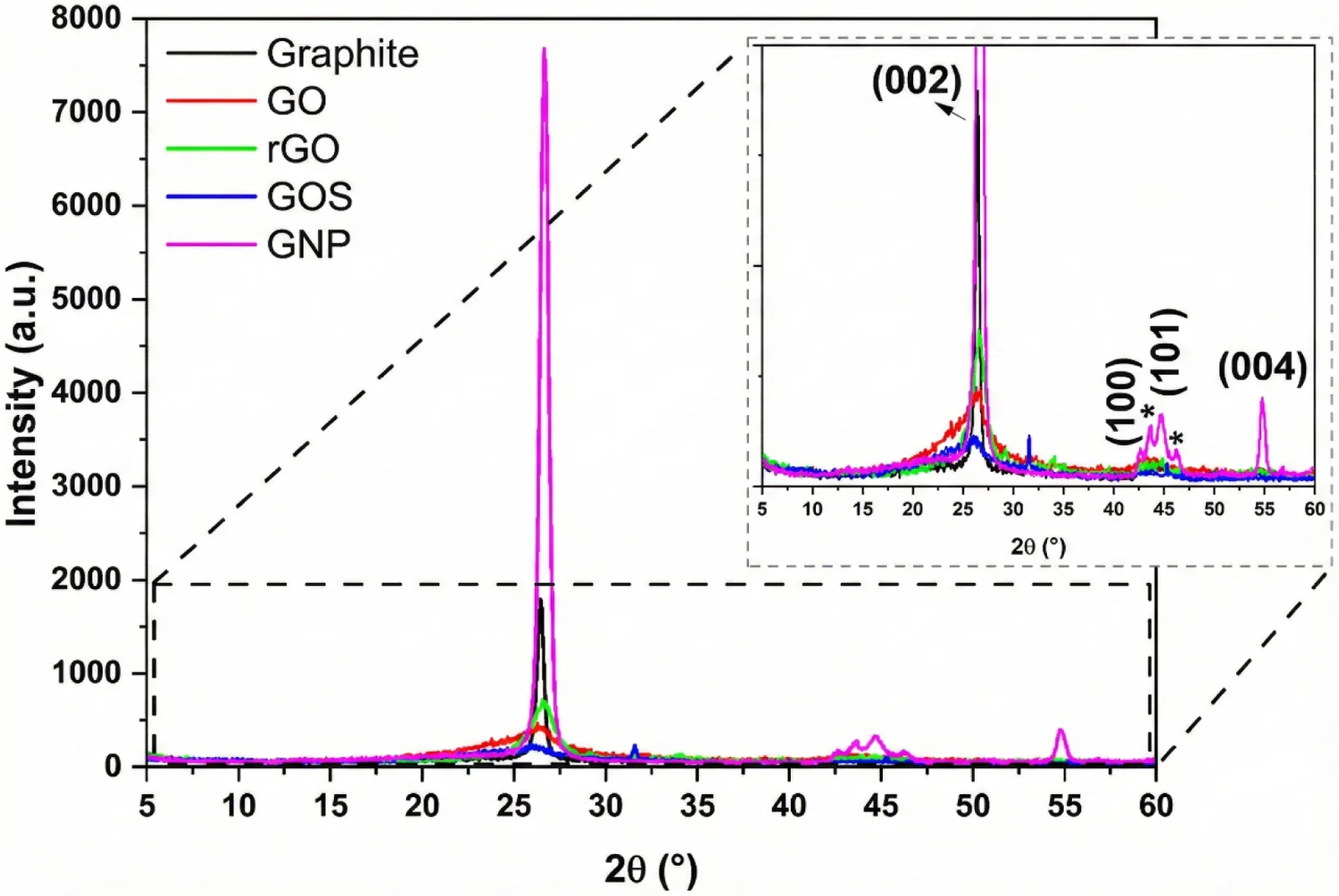
XRD patterns of graphite and graphene-based fillers (GO, rGO, GOS, and GNP) used as reinforcements in the PE-RT matrix. The inset shows the same 2θ range on an expanded intensity scale to highlight the weaker diffraction reflections.

#### 3.1.3 FTIR

The FTIR spectrum of GO exhibits a broad absorption band centered at approximately 3392 cm^–1^, attributed predominantly to O–H stretching vibrations associated with hydroxyl groups and adsorbed water. The band at 1728 cm^–1^ is assigned to C=O stretching in carbonyl and carboxyl-containing groups, whereas the absorption near 1070 cm^–1^ is associated with C–O–C/C–O vibrations of oxygen-containing functionalities.^28,29^ These bands confirm the introduction of oxygen-containing groups during graphite oxidation. In the rGO spectrum, the intensities of these oxygen-related bands decrease markedly, consistent with extensive deoxygenation during thermal reduction.^37^ For GOS, additional spectral changes are observed, particularly the feature near 1399 cm^–1^ and bands within the 1133–764 cm^–1^ region. These changes are consistent with the incorporation of Si-containing species derived from VTMS.^30^ However, because overlapping C–O, Si–O–C, and Si–O–Si vibrations may occur in this spectral region,^30^ the FTIR data alone are not considered unequivocal evidence of covalent grafting. The assignment is therefore supported by the EDS analysis, which detected 2.98 wt % Si in GOS (Figure 3).

**Figure 3:**
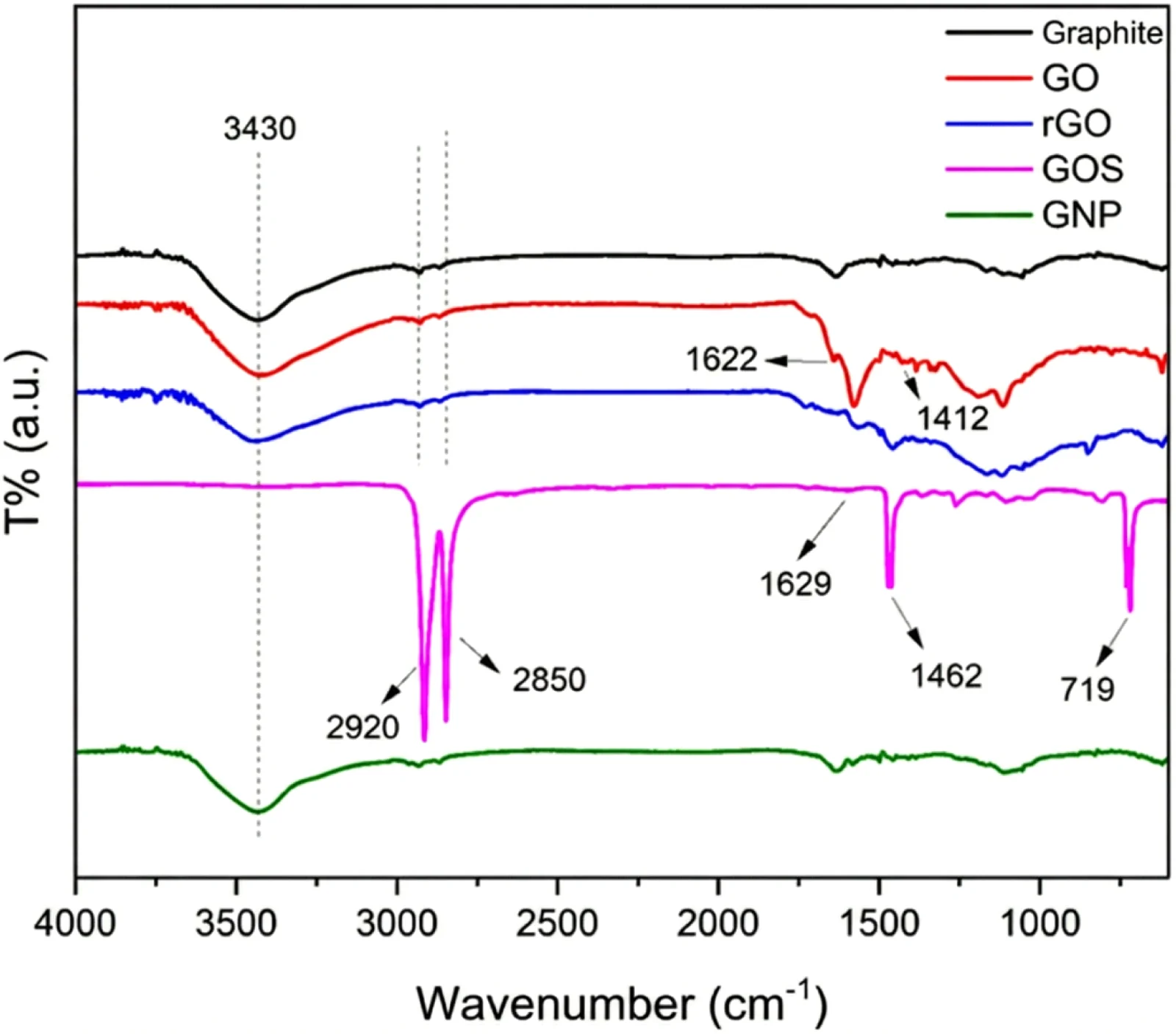
FTIR spectra of graphite and graphene-based fillers (GO, rGO, GOS, and GNP), with the main characteristic absorption bands indicated.

#### 3.1.4 Raman Spectroscopy

All samples display the characteristic D (≈1350 cm^–1^), G (≈1580 cm^–1^), and 2D (≈2700 cm^–1^) bands of graphitic carbon^38–40^ (Figure 4). Graphite shows a low I_D_/I_G_ ratio and comparatively sharp graphitic bands, consistent with its high crystalline order.^41^ GO exhibits a D band near 1340 cm^–1^ and an increased I_D_/I_G_ ratio, reflecting oxidation-induced structural disorder.^42^ rGO shows the highest I_D_/I_G_ ratio of the series (1.26), consistent with the increased defect density associated with high-temperature reduction and incomplete reconstruction of the graphitic domains.^43^ GOS exhibits D and G bands at 1339 and 1578 cm^–1^, respectively, with no appreciable shift relative to GO, and an I_D_/I_G_ ratio of 0.82. These results indicate that VTMS functionalization did not produce a measurable increase in Raman-detectable structural disorder and that the overall defect structure of GO was largely preserved.^30^ GNP exhibits comparatively sharp D and G bands and an I_D_/I_G_ ratio of 1.15, consistent with a significant contribution from edge defects in multilayer nanoplatelets.^32^ Thus, the Raman interpretation considers peak positions, band profiles, and relative intensities rather than relying exclusively on I_D_/I_G_. Raman spectroscopy alone, however, cannot establish the degree of exfoliation or nanoscale dispersion of the fillers.

**Figure 4:**
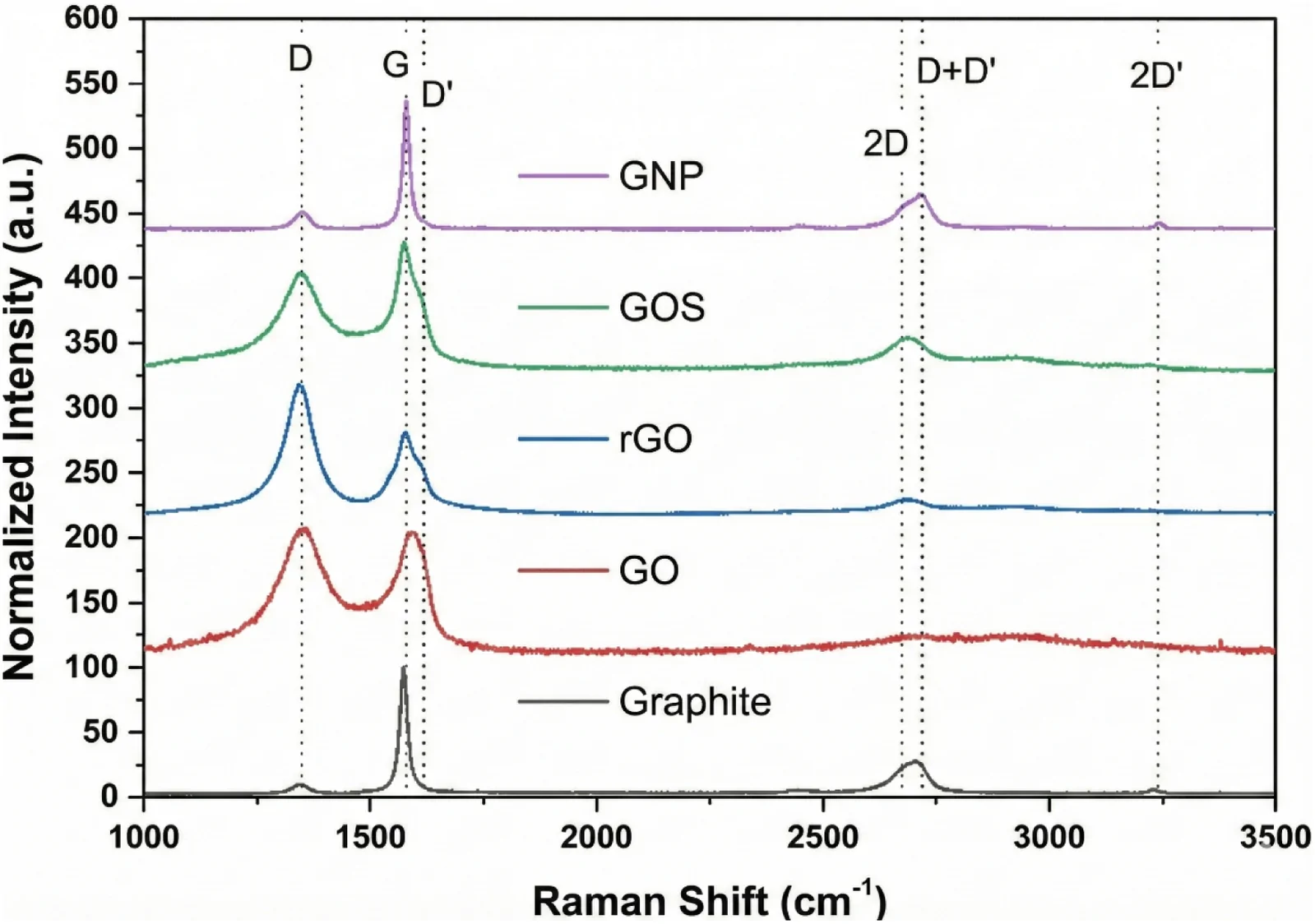
Raman spectra of graphite and graphene-based fillers (GO, rGO, GOS, and GNP), with the main characteristic Raman bands indicated.

The higher I_D_/I_G_ ratio observed for rGO is consistent with the structural changes expected during high-temperature thermal reduction. Although treatment at 1000 °C efficiently removes oxygen-containing functional groups, the rapid evolution of CO, CO_2_, and other gaseous species during deoxygenation can generate vacancies, edge defects, fragmentation, and smaller sp^2^ domains. Therefore, the increased I_D_/I_G_ ratio should not be interpreted solely as evidence of reduction, but also as an indication of increased structural disorder resulting from the severe thermal treatment. The SEM observations, which reveal thinner, wrinkled, and more fragmented rGO sheets compared with GO, further support this interpretation

#### 3.1.5 SEM and EDS

Graphite displays compact, highly stacked lamellar aggregates. GO shows irregular, overlapping platelets with increased apparent thickness due to oxygenated groups.^44^ rGO exhibits thinner, corrugated sheets with wrinkles and folds characteristic of thermal reduction.^45^ GOS exhibits a more open, sheet-like morphology, consistent with reduced apparent stacking, which may be associated with steric effects introduced by VTMS functionalization;^46^ EDS analysis of GOS revealed a Si content of 2.98 wt %, whereas Si was not detected in unmodified GO, supporting the incorporation of VTMS-derived species during the silanization treatment. GNP shows compact, flocculated multilayer platelets, consistent with its low defect content^47^ (Figures 5 and 6). GNP exhibits more compact platelet assemblies and greater apparent stacking at the microscale than the chemically modified graphene-based fillers. However, SEM provides only two-dimensional qualitative information on the examined fracture surfaces and cannot establish the three-dimensional extent or size distribution of GNP agglomerates within the PE-RT matrix. Therefore, the observed morphology is interpreted as being consistent with a greater tendency toward restacking/agglomeration rather than as quantitative evidence of filler aggregation. High-resolution TEM or three-dimensional imaging would be required to quantify the size, spatial distribution, and extent of GNP agglomerates. Because nanoscale filler dispersion within the PE-RT matrix was not directly resolved by SEM at the length scales examined, relationships between filler morphology and barrier, thermal, and mechanical performance are discussed throughout Sections 3.2–3.5 as structure–property interpretations rather than direct measurements of nanoscale dispersion.

**Figure 5:**
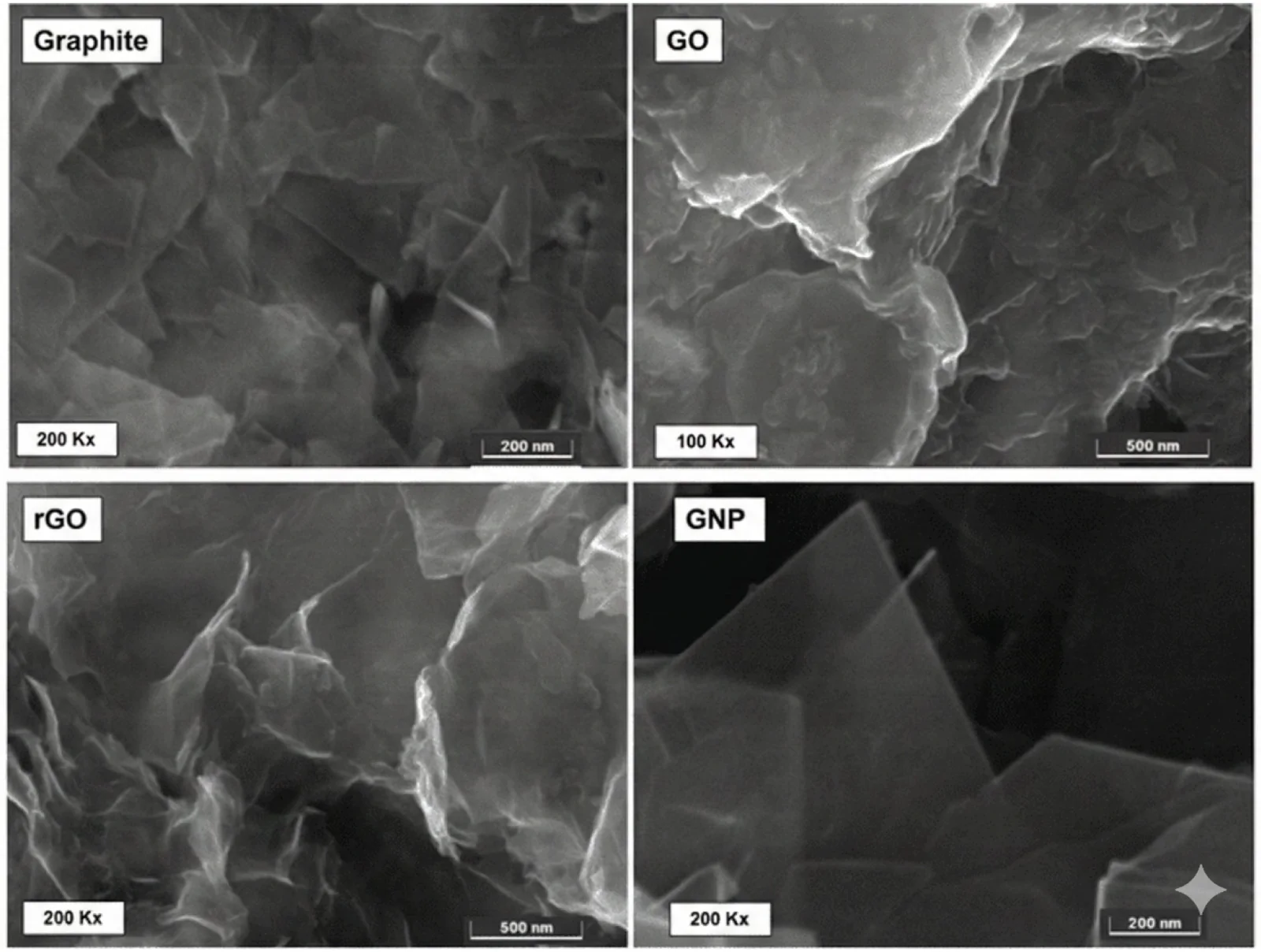
SEM micrographs of (a) graphite, (b) graphene oxide (GO), (c) reduced graphene oxide (rGO), and (d) commercial graphene nanoplatelets (GNP).

**Figure 6:**
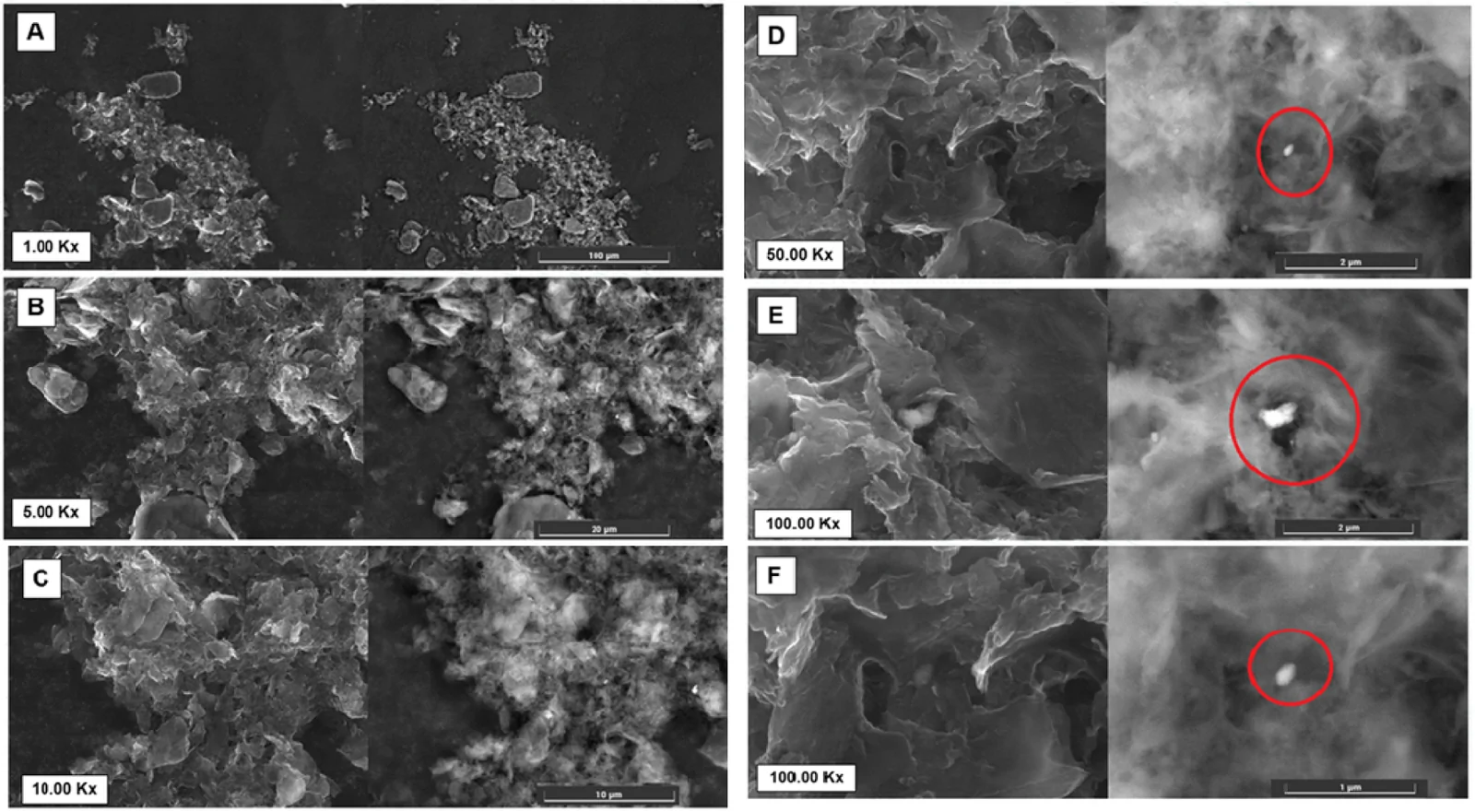
SEM images of the GOS filler at different magnifications and representative regions selected for point EDS analysis: (a) 1,000×, (b) 5,000×, (c) 10,000×, (d) 50,000×, and (e, f) 100,000×. The red circles in (d–f) indicate the specific regions selected for point EDS analysis.

### 3.2 Thermal Properties of Nanocomposites

#### 3.2.1 TGA

Neat PE-RT exhibited a single main degradation step, with *T*_10%_ = 431 ± 2 °C, *T*_onset_ = 446 ± 3 °C, and *T*_max_ = 474 ± 2 °C (Table 1 and Figure 7). Incorporation of the graphene-based fillers produced filler- and concentration-dependent changes in thermal stability. For GO-containing nanocomposites, *T*_10%_ and *T*_onset_ increased progressively with filler content, reaching 445 ± 3 and 458 ± 4 °C, respectively, at 2 wt % GO. GOS showed a similar trend, with the highest nominal *T*_onset_ value (461 ± 4 °C) was observed at 2 wt %. These increases may be associated with the ability of dispersed layered fillers to hinder the transport of volatile degradation products and with interfacial interactions between the filler and polymer matrix.^48,49^ In contrast, rGO produced only modest changes in *T*_onset_ over the investigated concentration range (451–453 °C). GNP also showed modest increases at 0.5 and 1 wt %, whereas at 2 wt % *T*_10%_, *T*_onset_, and *T*_max_ decreased to 427 ± 5, 440 ± 5, and 470 ± 5 °C, respectively. Because several of the observed differences are comparable to the experimental uncertainties, small shifts in degradation temperature should not be overinterpreted. Overall, the TGA results indicate that GO and GOS generally maintain or moderately increase the thermal stability of PE-RT, whereas the effect of rGO is smaller and the highest GNP loading adversely affects thermal stability.

**Table 1:** Results Obtained by TGA for Nanocomposites with GO, rGO, GOS, and GNP

SAMPLE	*T*_10%_ (°C)	*T*_onset_ (°C)	*T*_max_ (°C)	Residue (%)
Intrepid	431 ± 2	446 ± 3	474 ± 2	0.08
IGO0.5	438 ± 4	455 ± 3	475 ± 4	0.29
IGO1.0	442 ± 3	456 ± 3	477 ± 5	1.02
IGO2.0	445 ± 3	458 ± 4	479 ± 4	0.70
IrGO0.5	439 ± 4	453 ± 5	475 ± 5	0.20
IrGO1.0	436 ± 4	451 ± 4	474 ± 6	0.40
IrGO2.0	435 ± 5	451 ± 5	474 ± 3	0.20
IGOS0.5	441 ± 3	454 ± 4	477 ± 4	0.05
IGOS1.0	441 ± 5	455 ± 5	477 ± 6	0.60
IGOS2.0	449 ± 4	461 ± 4	482 ± 5	0.34
IGNP0.5	438 ± 5	452 ± 4	475 ± 5	0.52
IGNP1.0	440 ± 4	455 ± 4	477 ± 5	0.56
IGNP2.0	427 ± 5	440 ± 5	470 ± 5	1.52

**Figure 7:**
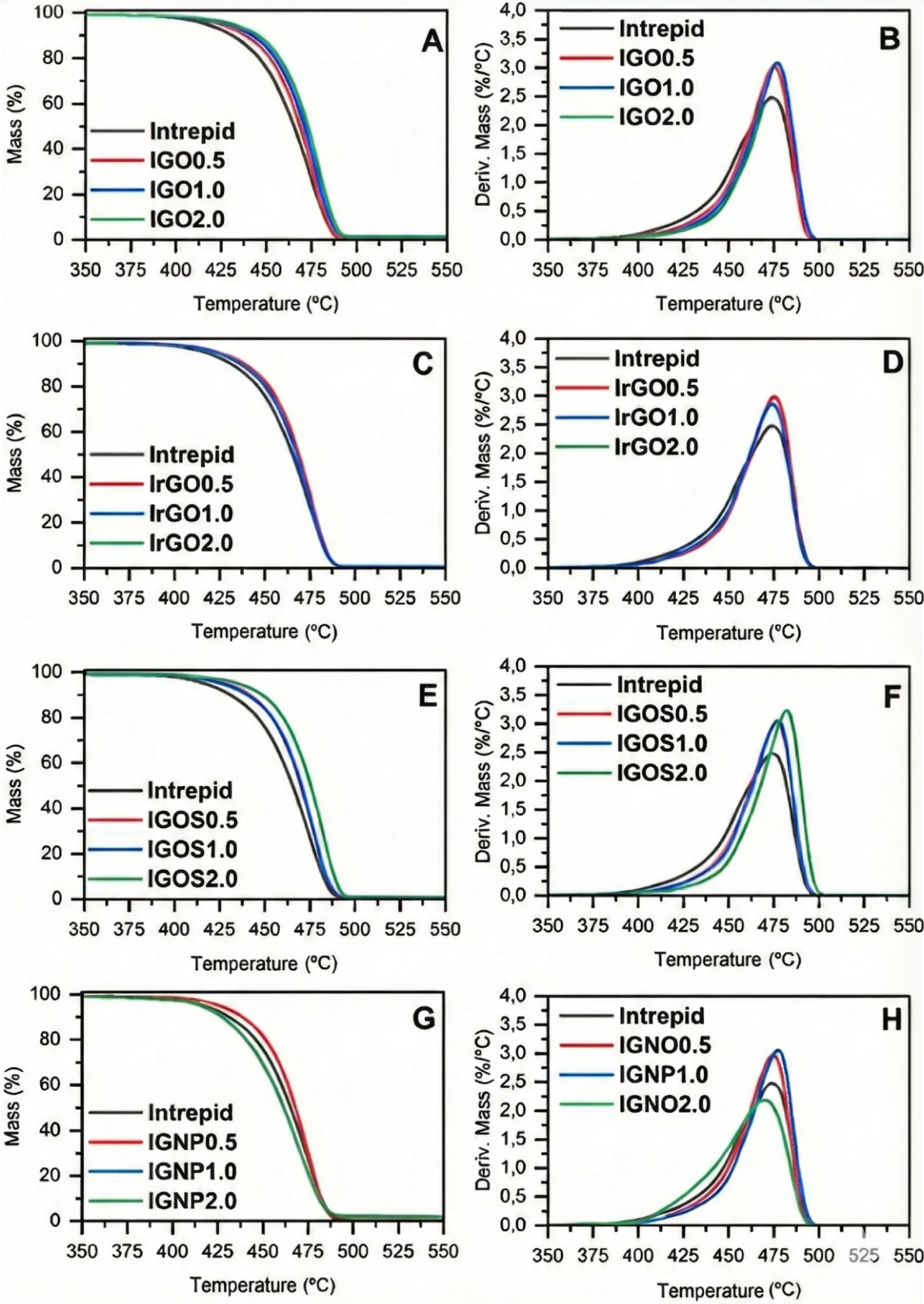
TGA and DTG curves of neat PE-RT (Intrepid) and graphene-based nanocomposites at different filler loadings: (a, b) IGO, (c, d) IrGO, (e, f) IGOS, and (g, h) IGNP, where (a, c, e, g) show the TGA curves and (b, d, f, h) the corresponding DTG curves.

#### 3.2.2 DSC

The incorporation of graphene-based fillers produced only minor changes in the melting and crystallization temperatures of PE-RT (Table S2). Across all formulations, *T*_m_ remained within 129–131 °C and *T*_c_ within 116–118 °C, indicating that the characteristic melting and crystallization transitions of the PE-RT matrix were largely preserved after nanofiller incorporation. More noticeable variations were observed in the degree of crystallinity (*X*_c_). At 0.5 wt %, GO and rGO produced the largest increases in *X*_c_ relative to neat PE-RT, consistent with a heterogeneous nucleation effect of the graphene-based surfaces.^50^ At higher filler contents, however, *X*_c_ varied nonmonotonically and generally approached the value of the neat polymer. GOS and GNP also produced relatively small, concentration-dependent changes in *X*_c_, with increases at lower or intermediate loadings followed by a decrease at 2 wt %. Such nonmonotonic behavior may reflect the competing effects commonly observed in polymer nanocomposites, in which nanofiller surfaces can provide heterogeneous nucleation sites while increasing filler content may restrict polymer-chain organization or reduce the effectiveness of individual particles as nucleating surfaces through aggregation.^50,51^ Overall, the DSC results show that the graphene-based fillers modify the extent of PE-RT crystallization to a limited and formulation-dependent degree without substantially changing *T*_m_ or *T*_c_. Moreover, the variations in *X*_c_ do not parallel the CO_2_ permeability trends discussed in Section 3.4, indicating that changes in bulk crystallinity alone cannot account for the observed barrier performance.

### 3.3 Mechanical Properties of Nanocomposites

#### 3.3.1 Shore D Hardness

The neat PE-RT matrix shows a Shore D hardness of 54 ± 1, consistent with its semicrystalline nature. GO and rGO yield consistent hardness gains across all loadings, reaching maximum increases of +3 (GO, 0.5 wt %) and +5 (rGO, 1 wt %) units. These hardness changes may reflect the combined effects of filler-induced changes in the semicrystalline microstructure and local polymer–filler interactions. GOS improves hardness at 1 wt %, but less markedly than GO and rGO, possibly because the VTMS layer partially shields the graphitic surface. GNP shows only a slight improvement at 0.5 wt %, followed by a decline at higher loadings, indicating a nonmonotonic concentration dependence. This behavior may reflect changes in platelet organization and polymer–filler interactions at increasing filler contents, although nanoscale aggregation was not directly quantified (Figure 8).

**Figure 8:**
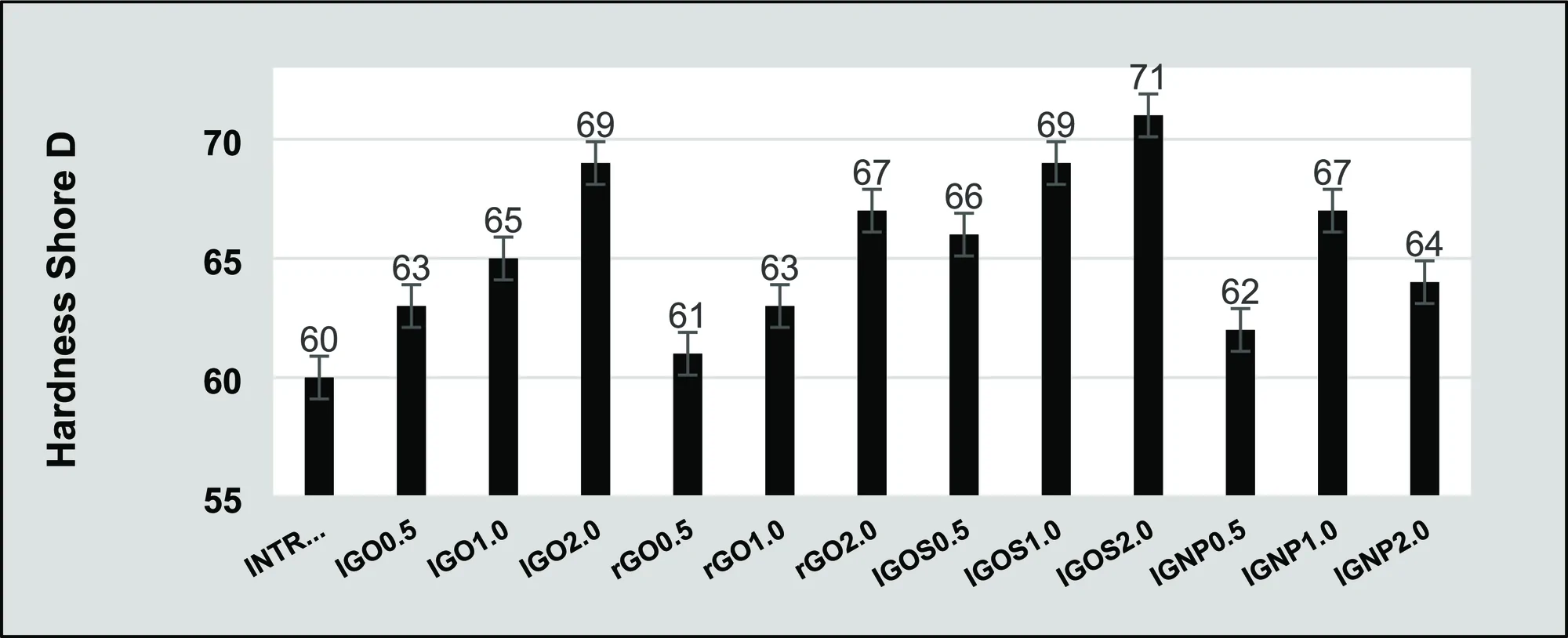
Shore D hardness of neat PE-RT (Intrepid) and nanocomposites containing GO, rGO, GOS, and GNP at different filler loadings (0.5, 1.0, and 2.0 wt %). Error bars represent the standard deviation.

#### 3.3.2 Tensile Properties

Tensile characterization focused on the 1 wt % formulations to allow direct comparison of the mechanical response of PE-RT containing the different graphene-based fillers at the same intermediate loading. Neat PE-RT exhibited a yield strength of 18.2 ± 1.3 MPa and an elastic modulus of 301.3 ± 31.7 MPa (Table S3). At 1 wt %, GO, rGO, GOS, and GNP exhibited mean elastic moduli of 410.0 ± 54.6, 348.4 ± 63.8, 376.4 ± 12.9, and 454.5 ± 30.7 MPa, respectively. One-way ANOVA revealed a significant effect of nanofiller type on elastic modulus (*p* < 0.001). GNP exhibited the highest mean stiffness, corresponding to an increase of approximately 50.8% relative to neat PE-RT, and this increase was statistically significant (Tukey’s HSD, *p* < 0.001). GO and GOS also exhibited significantly higher elastic moduli than neat PE-RT (*p* < 0.05), whereas the increase observed for rGO was not statistically significant (*p* > 0.05). Although GNP showed the highest mean modulus, its value was not significantly different from that of GO (*p* > 0.05). Thus, all four graphene-based fillers increased the mean stiffness and yield strength of PE-RT at 1 wt %, although the magnitude of the response depended on filler type. These results also indicate that the factors governing mechanical reinforcement differ from those controlling gas-barrier performance, since GNP exhibited the highest mean mechanical properties despite its comparatively poorer barrier performance.^52–54^

### 3.4 CO_2_ Barrier Performance and Nielsen Model Analysis

The CO_2_ permeability coefficients of neat PE-RT and its nanocomposites at 1 wt % are summarized in Table 2 and Figure 9. The neat polymer exhibits *K* = 4.90 ± 0.10 cm^2^·s^–1^·psi^–1^.

**Table 2:** CO_2_ Permeability Coefficients of Neat PE-RT and Nanocomposites at Different Nanofiller Contents^a^

SAMPLE	Coefficient of Permeability (cm^2^ s^–1^ psi^–1^)	Time Lag (h)	Coefficient of Diffusibility 10^3^ (cm^2^/s)
Intrepid	4.90 ± 0.10	0.79 ± 0.3	4.90 ± 0.8
IGO1.0	6.90 ± 0.12	3.14 ± 0.2	7.00 ± 0.7
IrGO1.0	5.70 ± 0.25	6.80 ± 0.5	5.80 ± 0.6
IGOS1.0	2.13 ± 0.30	13.15 ±0.3	1.40 ± 0.2
IGNP1.0	6.00 ± 0.15	5.37 ± 0.5	5.84 ± 0.5

a Values are reported as mean ± standard deviation (n = 3; independent specimens).

**Figure 9:**
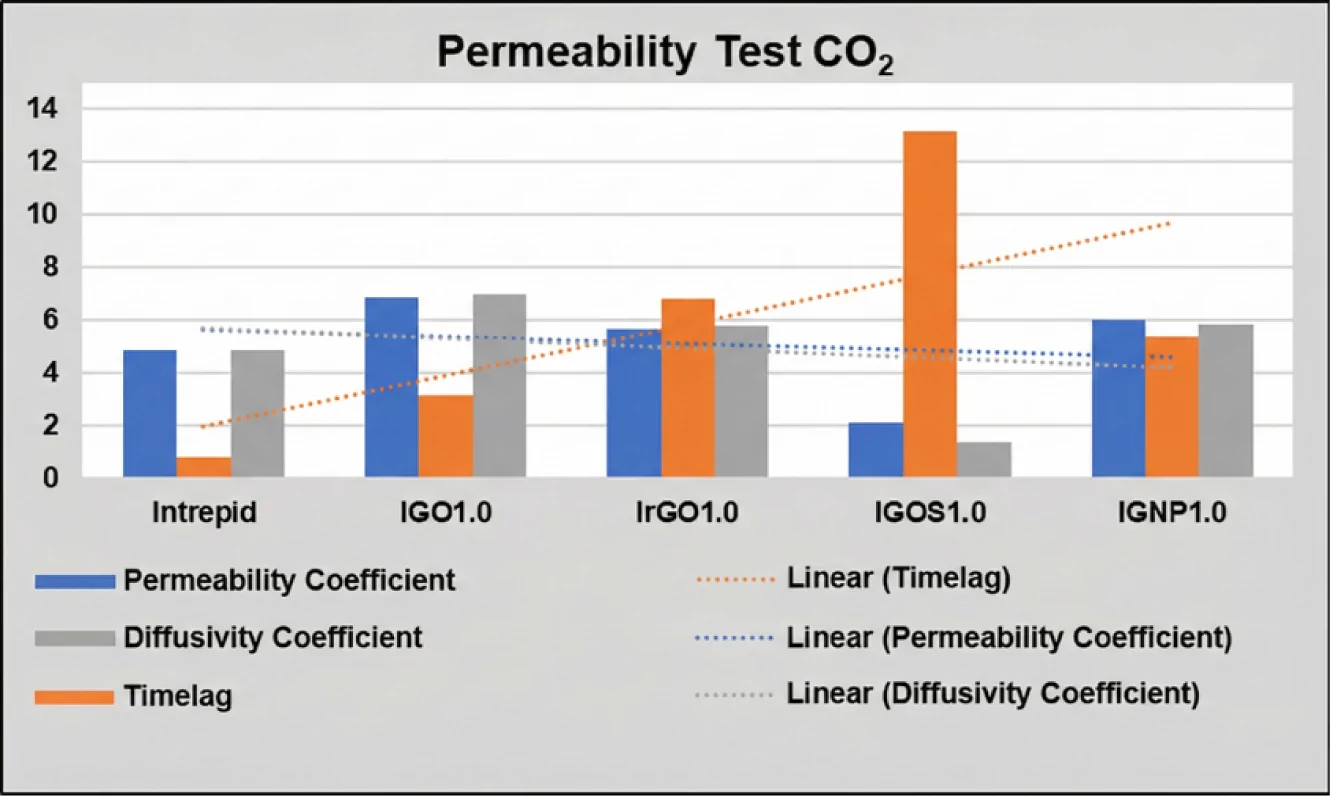
CO_2_ permeability coefficient, time lag, and diffusivity coefficient for neat PE-RT (Intrepid) and nanocomposites containing 1.0 wt % GO, rGO, GOS, and GNP. Data are presented as mean ± standard deviation (*n* = 3 independent specimens).

GO and rGO both increase permeability relative to neat PE-RT (Table 2). For GO, the high oxygen functional group content interferes with chain packing, increasing the free volume available for gas transport despite the expected tortuosity effect. For rGO, the reduced functionality improves sheet rigidity, but insufficient dispersion at 1 wt % limits the barrier effect.

GOS at 1 wt % (IGOS1.0) produced the greatest CO_2_ barrier improvement, reducing the mean permeability by 56.5% relative to neat PE-RT (2.13 ± 0.30 vs 4.90 ± 0.10 cm^2^ s^–1^ psi^–1^; Tukey HSD, *p* < 0.001). This improvement was accompanied by an increase in diffusion time lag from 0.79 ± 0.3 to 13.15 ± 0.3 h and a reduction in diffusion coefficient from (4.90 ± 0.8) × 10^–3^ to (1.40 ± 0.2) × 10^–3^ cm^2^ s^–1^.^55^ This remarkable improvement cannot be attributed to crystallinity alone: IGOS1.0 shows only modest Xc variation (48% vs 49% for neat PE-RT, Table S2), whereas IGNP1.0 (*X*_c_ = 56%) shows increased permeability, probably due to aggregation. The superior barrier response of GOS should not be attributed to dispersion alone. Rather, the experimental results are consistent with the combined influence of several factors introduced or affected by VTMS functionalization. First, silane functionalization modifies the surface chemistry of GO and may improve compatibility with the nonpolar PE-RT matrix, thereby affecting polymer–filler interfacial interactions. Second, the more open sheet-like morphology observed for GOS by SEM is consistent with reduced apparent platelet stacking before incorporation into the polymer; however, nanoscale exfoliation within the PE-RT matrix was not directly quantified. Third, platelet orientation during melt processing was not experimentally determined and therefore its specific contribution to gas transport cannot be isolated. Finally, the Nielsen analysis indicates that a substantial platelet-induced tortuosity is required to reproduce the measured permeability reduction. Thus, the 56.5% decrease in CO_2_ permeability is best interpreted as the combined outcome of GOS surface chemistry, polymer–filler interfacial characteristics, effective platelet geometry and arrangement, and the resulting tortuous diffusion pathways, rather than as a consequence of improved dispersion alone.

To examine the contribution of platelet-induced tortuosity, the Nielsen model was applied according to

$$P/P_{0}=\left(1-\varphi \right)/\left[1+\left(\alpha /2\right)\varphi \right]$$

Where *P* and *P*_0_ are the permeabilities of the nanocomposite and neat polymer, respectively, φ is the filler volume fraction, and α is the platelet aspect ratio. For IGOS1.0, φ = 0.0045 was calculated from the nominal filler mass fraction using densities of 2.1 g cm^–3^ for GOS and 0.945 g cm^–3^ for PE-RT. The calculation assumes impermeable platelets with an effective orientation favorable to the barrier effect, homogeneous filler distribution at the continuum scale, and no explicit contribution from interfacial voids or changes in polymer-chain mobility. Under these assumptions, rearrangement of the Nielsen equation using the experimentally measured permeability ratio (*P*/*P*_0_ ≈ 0.43) and the calculated filler volume fraction (φ = 0.0045) yields an effective aspect ratio, α, of approximately 570. This value was not obtained from direct measurements of platelet lateral dimensions or thickness; rather, it represents a model-derived effective transport parameter corresponding to the platelet geometry and orientation required within the Nielsen framework to reproduce the experimentally observed permeability reduction. Because platelet dimensions and thickness were not independently quantified by TEM, AFM, or other nanoscale imaging methods, an α of approximately 570 should not be interpreted as an experimentally measured geometrical aspect ratio or as a direct measure of exfoliation or nanoscale dispersion.

Because the Nielsen prediction is sensitive to platelet geometry and orientation, a sensitivity analysis was performed over a range of aspect ratios (Table S4). The predicted relative permeability decreases progressively with increasing α, illustrating that the magnitude of the calculated barrier effect strongly depends on the effective platelet geometry assumed in the model. Accordingly, the Nielsen analysis is used here to estimate the level of platelet-induced tortuosity required to reproduce the experimental barrier response rather than to determine the actual morphology of GOS within the PE-RT matrix.

The markedly lower permeability of IGOS1.0 therefore indicates that VTMS functionalization favors a filler–matrix configuration that is more effective in restricting CO_2_ transport than those obtained with the other graphene-based fillers. This interpretation is consistent with the substantially longer time lag and lower diffusion coefficient measured for IGOS1.0, indicating slower CO_2_ transport through the nanocomposite. However, because nanoscale dispersion and platelet orientation were not directly quantified, the relative contributions of platelet arrangement, interfacial interactions, and polymer-matrix organization cannot be independently resolved from the present data. In contrast, GO, rGO, and GNP did not produce a comparable barrier improvement at 1 wt %, despite differences in crystallinity and mechanical response. Taken together, these results show that bulk crystallinity alone cannot explain the observed permeability trends and support an important role of filler surface chemistry and effective transport-path tortuosity in controlling CO_2_ permeation in PE-RT nanocomposites.

The assumptions and limitations of the Nielsen model should be considered in relation to the distinct characteristics of the graphene-based fillers investigated here. The model assumes impermeable platelets with an effective orientation favorable for the barrier effect and a homogeneous distribution within the polymer matrix. The filler volume fraction (φ) was calculated from the nominal mass fraction and the assumed densities of the filler and PE-RT and, therefore, does not account for possible local concentration variations. The effective aspect ratio (α) is a model-derived transport parameter rather than a direct measurement of platelet dimensions. In addition, the model does not explicitly account for filler aggregation or restacking, interfacial voids, or changes in polymer-chain mobility, all of which may alter the effective diffusion pathways. These assumptions may not be equally applicable to GO, rGO, GOS, and GNP because differences in surface chemistry, platelet stacking, orientation, and interfacial interactions may produce filler-specific transport behavior. Consequently, the Nielsen analysis is used here primarily as a comparative framework to estimate the level of platelet-induced tortuosity required to reproduce the experimental barrier response of GOS, rather than as a direct representation of filler morphology or a complete description of gas transport in all four nanocomposite systems.

### 3.5 Surface Wettability

The neat PE-RT matrix shows a contact angle of 97.5°, indicating moderate hydrophobicity.^56^ GO incorporation increases the contact angle progressively to 102° at 2 wt %, associated with changes in surface characteristics induced by GO incorporation, which may be advantageous for limiting water uptake in riser applications.^57^ rGO shows a slight, concentration-independent increase, reflecting its graphitic character. GOS yields the highest contact angles in the series (103° at 1 wt %, 106° at 2 wt %), in line with the reduced polarity imparted by VTMS functionalization. GNP shows a slight decrease in contact angle at 2 wt %, indicating a formulation-dependent change in surface wettability. Because contact angle can be influenced by both surface chemistry and surface roughness,^58^ and because these surface characteristics were not independently quantified, this variation cannot be specifically attributed to filler aggregation. Overall, GOS exhibits the highest contact angles among the investigated formulations, consistent with the reduced surface polarity associated with VTMS modification. Accordingly, the contact-angle results are interpreted as indicators of surface wettability rather than as a direct measure of filler dispersion or interfacial adhesion (Figure 10).

**Figure 10:**
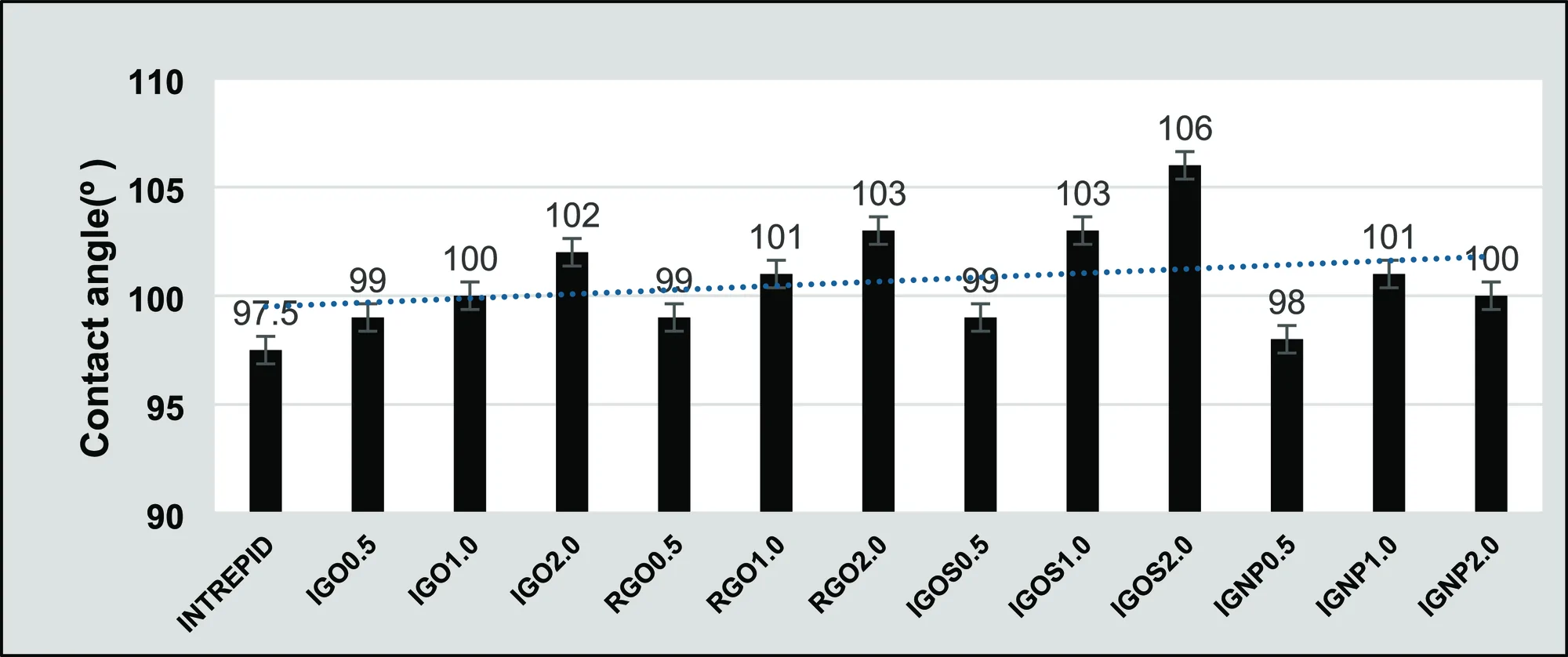
Water contact angle of neat PE-RT (Intrepid) and nanocomposites containing 0.5, 1.0, and 2.0 wt % GO, rGO, GOS, and GNP. Values represent the mean ± standard deviation of four measurements per sample.

### 3.6 Aging Performance

#### 3.6.1 Visual Inspection and Swelling

The neat PE-RT matrix exhibited progressive yellowing from the second month and pronounced warping by the fourth month, indicating substantial macroscopic changes during prolonged hydrocarbon exposure at 80 °C.^59–61^ In contrast, all nanocomposites retained their original color throughout the experiment and exhibited only minor dimensional changes. After 6 months, the degree of swelling reached 11.2% for neat PE-RT, compared with 4.5% for IGO1.0, 6.8% for IrGO1.0, and 4.1% for IGOS1.0. Thus, among the investigated nanocomposites, IGOS1.0 exhibited the lowest swelling, whereas IrGO1.0 showed the highest. These differences indicate a filler–matrix-dependent response to hydrocarbon uptake, although the respective contributions of filler–matrix interactions, morphology, and transport pathways cannot be independently resolved based on the present data (Figure 11).

**Figure 11:**
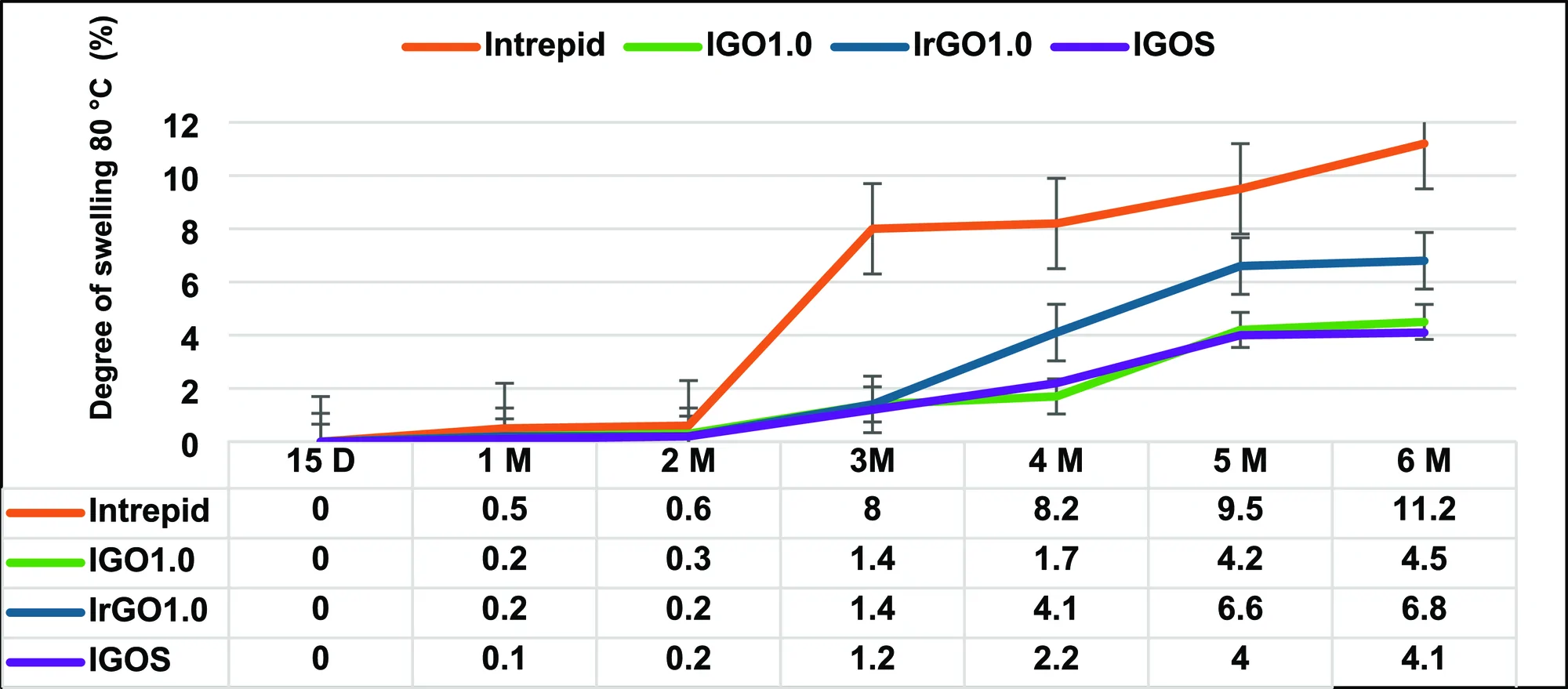
Degree of swelling of neat PE-RT (Intrepid) and nanocomposites containing 1.0 wt % GO, rGO, and GOS during aging in synthetic oil at 80 °C for 15 days and 1–6 months. Values are presented as mean ± standard deviation.

#### 3.6.2 TGA after Aging

TGA of aged nanocomposites (Figure 12, Table S5, Figure S1) reveals markedly different degradation behavior compared with the neat polymer.

**Figure 12:**
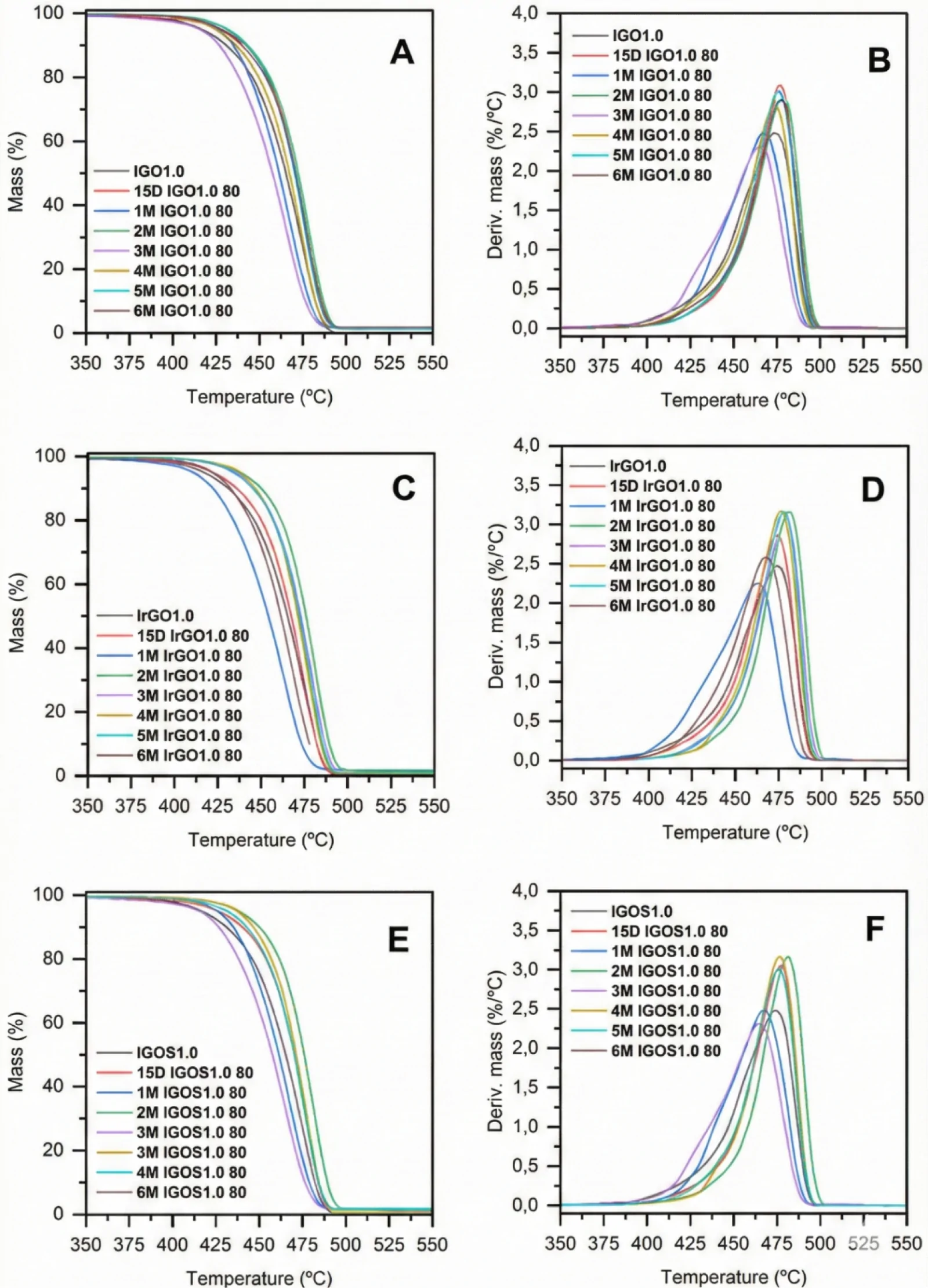
TGA and DTG curves of PE-RT/graphene nanocomposites before and after aging at 80 °C for 15 days and 1–6 months: (a, b) IGO1.0, (c, d) IrGO1.0, and (e, f) IGOS1.0, where (a, c, e) show the TGA curves and (b, d, f) the corresponding DTG curves.

After 6 months, IGO1.0 retained *T*_onset_ = 455 °C (virtually unchanged from the unaged value), remaining 9 °C above the unaged neat PE-RT baseline (446 °C). IrGO1.0 and IGOS1.0 maintained *T*_onset_ = 443 °C at 6 months, comparable to the neat polymer’s unaged value. In contrast, the unfilled matrix underwent severe thermal deterioration as evidenced by pronounced yellowing and warping. The nonmonotonic fluctuations in *T*_onset_ over the aging period (standard deviations of 7.4, 9.9, and 8.5 °C for IGO1.0, IrGO1.0, and IGOS1.0, respectively) reflect competing processes: initial solvent-induced plasticization followed by partial crystalline reorganization and interfacial strengthening. Within the measurement uncertainty (±5 °C, Table S6), the overall conclusion is that thermal stability was preserved in all nanocomposites throughout the test period.

The 6-month immersion protocol at 80 °C in synthetic hydrocarbon oil, conducted according to ISO 23936-1, provides a controlled accelerated assessment of material response to prolonged hydrocarbon exposure at elevated temperature. However, these conditions reproduce only selected aspects of the complex environment experienced by polymer barrier layers in offshore flexible risers and should not be interpreted as a direct simulation of field service life. In operation, the polymer may be simultaneously exposed to elevated pressure, dissolved gases such as CO_2_, water, temperature fluctuations, and sustained or cyclic mechanical stresses, which may alter fluid transport, oxidation, swelling, chain mobility, and damage evolution. Furthermore, the 6-month exposure period does not reproduce the substantially longer service times expected for offshore components. The present aging results should therefore be interpreted as a comparative assessment of the relative stability of the investigated formulations under standardized hydrocarbon exposure rather than as a prediction of long-term field lifetime. Validation under combined pressure, gas/water exposure, thermal cycling, mechanical loading, and longer aging periods will be required before extrapolating these results to actual flexible-riser service conditions.

Considering the structural, barrier, mechanical, and aging results together, no single descriptor showed a direct quantitative correlation with all measured properties. In particular, the Raman I_D_/I_G_ ratio and XRD structural parameters characterize different aspects of filler structure but do not independently predict CO_2_ permeability or mechanical reinforcement. Likewise, the effective aspect ratio of ≈570 obtained for IGOS1.0 from the Nielsen model is a transport-derived parameter specific to the GOS barrier response and cannot be treated as an independently measured morphological variable for correlation across all formulations. The available data set therefore does not support a statistically meaningful multivariate correlation among I_D_/I_G_, *d*-spacing, aspect ratio, permeability, modulus, and aging retention. Instead, comparison of the experimentally measured responses identifies IGOS1.0 as the formulation providing the best overall balance of properties: it produced the largest CO_2_ permeability reduction (56.5%), increased the mean elastic modulus relative to neat PE-RT, and showed comparatively stable behavior during 6 months of hydrocarbon aging. GNP at 1 wt % provided the highest mean elastic modulus but showed comparatively poor CO_2_ barrier performance. Thus, GOS at 1 wt % represents the optimum formulation among those investigated when barrier, mechanical, and aging performance are considered jointly, whereas GNP at 1 wt % is preferred when stiffness is considered independently.

#### 3.6.3 DSC after Aging

Melting and crystallization temperatures remained essentially unchanged throughout the 6-month period (*T*_m_ varied by only 3 °C across all samples, *T*_c_ by only 1 °C; Table S7, Figure S2), confirming that oil immersion did not significantly alter the crystalline phase transitions of PE-RT or its nanocomposites. Although modest fluctuations in Xc occurred (Figure 13, Table S7), no systematic reduction was detected, indicating satisfactory preservation of the semicrystalline microstructure, particularly in the graphene-containing nanocomposites, which exhibited comparatively greater retention of semicrystalline order during aging.^19,62^

**Figure 13:**
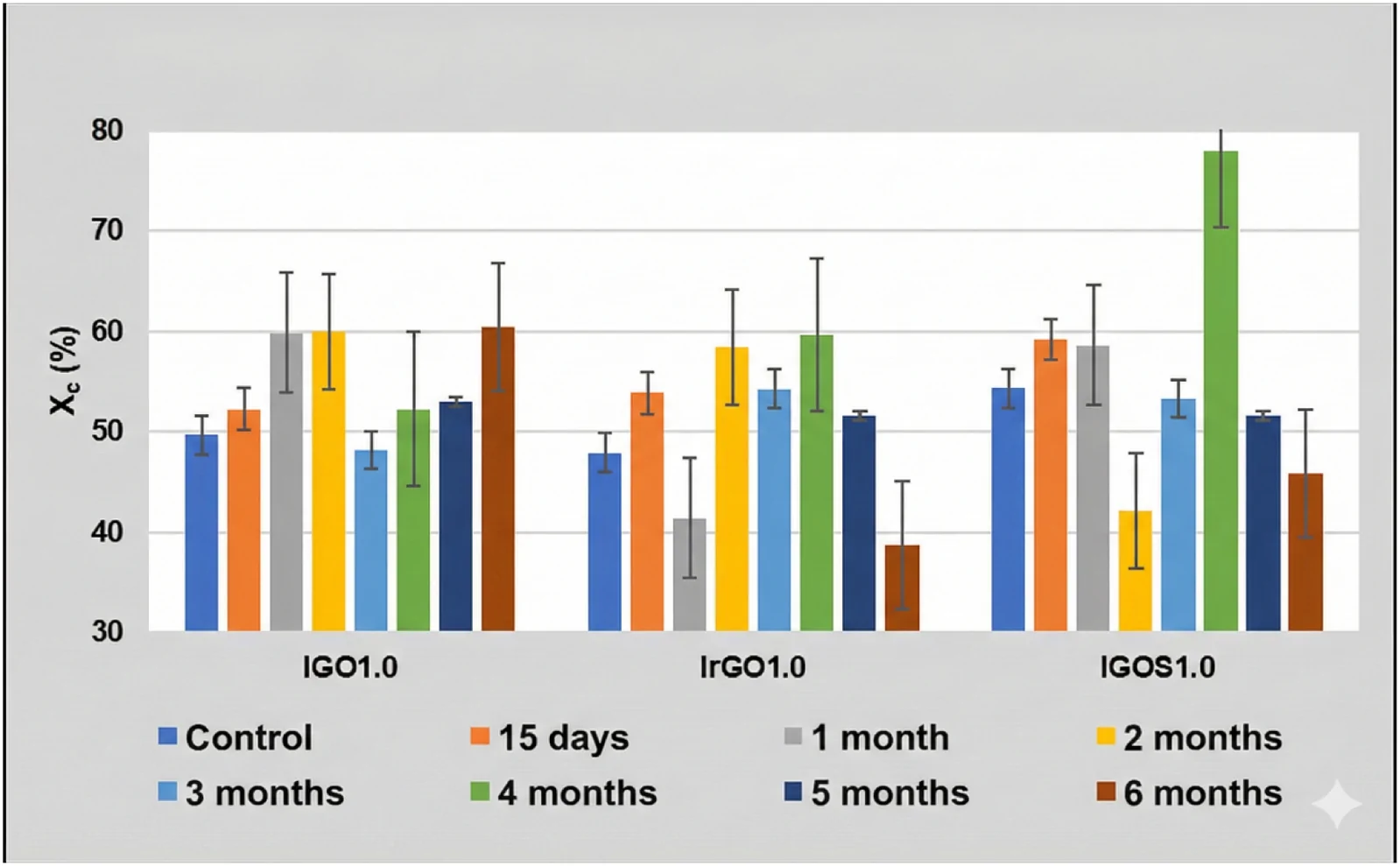
Degree of crystallinity (*X*_c_) determined by DSC for IGO1.0, IrGO1.0, and IGOS1.0 nanocomposites before and after aging in synthetic oil at 80 °C for 15 days and 1–6 months. Values are presented as mean ± standard deviation.

#### 3.6.4 Contact Angle after Aging

After 6 months, the neat PE-RT matrix showed a 5° reduction in contact angle, indicating increased wettability and susceptibility to moisture ingress. Nanocomposites exhibited substantially smaller changes: GO- and rGO-reinforced samples showed only −2° variation, while GOS showed only – 1°, indicating that graphene-based fillers mitigate aging-induced increases in surface energy, contributing to extended barrier durability (Figure 14).

**Figure 14:**
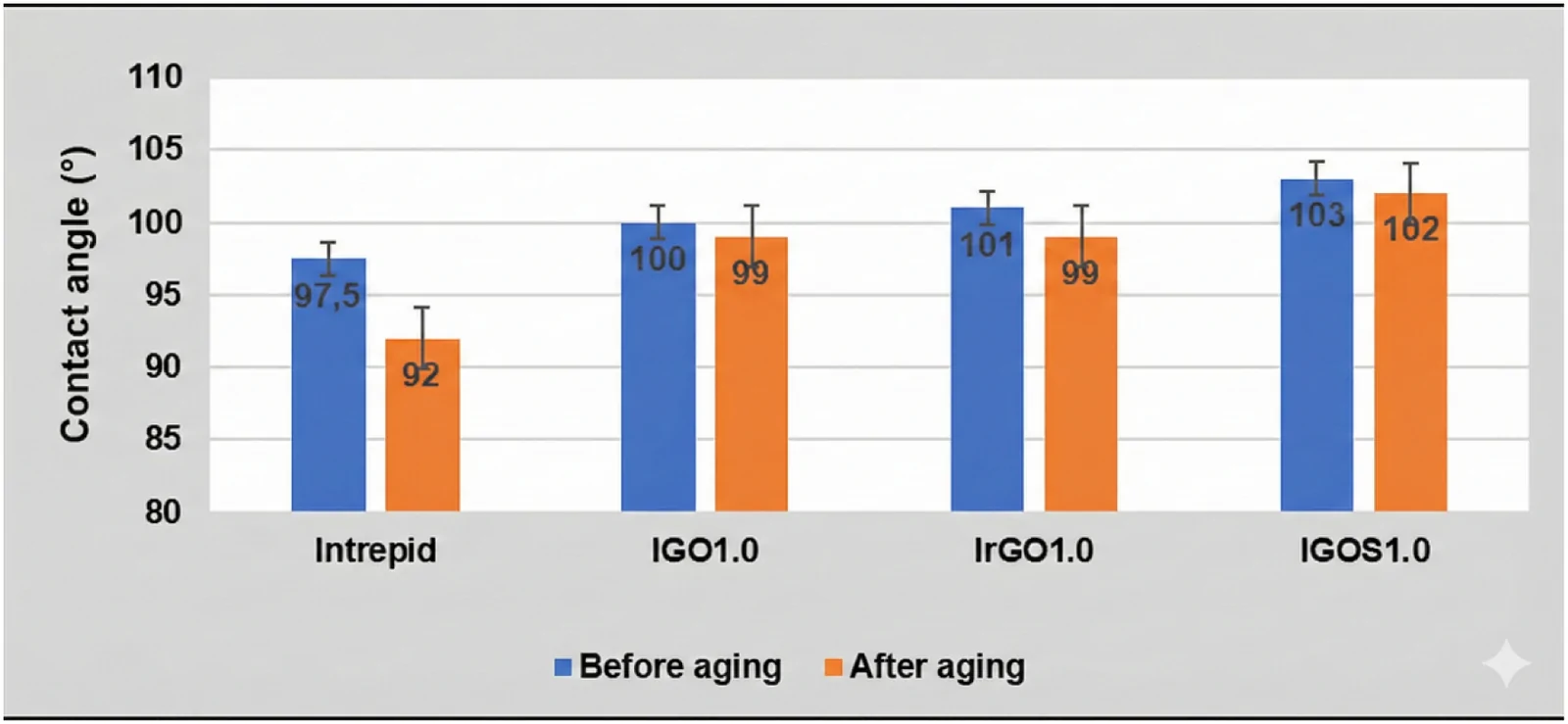
Water contact angle of neat PE-RT (Intrepid) and nanocomposites containing 1.0 wt % GO, rGO, and GOS before and after 6 months of aging in synthetic oil at 80 °C. Values are presented as mean ± standard deviation.

#### 3.6.5 GPC after Aging

GPC analysis revealed formulation-dependent changes in the molar-mass distributions during aging (Table S8 and Figure S3). For IGO1.0, *M*_n_ changed from 32 × 10^3^ to 29 × 10^3^ after 6 months, whereas *M*_v_ decreased from 546 × 10^3^ to 269 × 10^3^ and *Đ* decreased from 17 to 9. IrGO1.0 showed a comparatively small change in *M*_n_, from 33 × 10^3^ to 31 × 10^3^, while *M*_v_ changed from 221 × 10^3^ to 288 × 10^3^ and *Đ* from 6 to 9. IGOS1.0 exhibited the greatest apparent retention of the high-molar-mass distribution, with *M*_n_ changing from 26 × 10^3^ to 25 × 10^3^, *M*_v_ from 493 × 10^3^ to 520 × 10^3^, and *Đ* from 18 to 21 after 6 months. These results indicate that the molar-mass evolution during aging was strongly formulation-dependent and that the previous generalized statement of preserved molar-mass integrity was overly broad. Because the analyses were performed using high-temperature GPC under conditions intended to fully dissolve the PE-RT samples, the measured distributions were not obtained from a preselected soluble fraction. Nevertheless, as with any molar-mass analysis of aged polyolefins, very extensive cross-linking could, in principle, impair complete dissolution and thereby limit characterization. No such separation into insoluble material was applied in the present study.

#### 3.6.6 Mechanistic Interpretation

The improved aging resistance observed for the GO- and GOS-based nanocomposites may result from complementary physical and chemical effects. Graphene-based platelets can increase the tortuosity of molecular diffusion pathways; however, the CO_2_ barrier behavior measured in this study cannot be directly extrapolated to O_2_ transport, because gas permeability depends on gas-specific solubility and diffusivity within the polymer matrix. Therefore, although restriction of oxygen transport by graphene-based platelets represents a possible contribution to oxidative stabilization, oxygen permeability was not measured in the present study, and this mechanism cannot be experimentally confirmed from the CO_2_ permeability results. Radical-scavenging activity associated with graphene-based materials may also contribute to stabilization, but this mechanism was likewise not directly investigated. Accordingly, the comparatively smaller changes observed in TGA, DSC, swelling, contact angle, and GPC measurements for the GO- and GOS-containing nanocomposites are interpreted as evidence of enhanced overall aging stability rather than as direct proof of either an oxygen barrier or radical-scavenging mechanism. The aging process may involve multiple concurrent phenomena, including oxidation, chain scission, cross-linking, additive extraction, and morphological reorganization, whose individual contributions cannot be distinguished from the present data set. Although DSC was used to monitor changes in thermal transitions and crystallinity during aging, quantitative FTIR carbonyl index analysis, mechanical property retention measurements, and postaging morphological characterization were not performed. Therefore, the present results are interpreted in terms of overall property retention during hydrocarbon aging rather than as a definitive identification of the underlying degradation mechanisms. The relative contributions of physical diffusion-barrier effects and chemical stabilization should be investigated through dedicated O_2_ permeability and oxidative aging measurements in future work.

## 4 Conclusions

This work establishes an integrated structure–property–performance framework for PE-RT nanocomposites reinforced with GO, rGO, GOS, and GNP and provides experimentally grounded insights for the development of graphene-functionalized polyolefin barrier materials. The following principal conclusions are drawn:

(1) GOS produced the most favorable CO_2_ barrier response among the investigated graphene-based fillers. At 1 wt %, GOS reduced CO_2_ permeability by 56.5% despite exhibiting a degree of crystallinity similar to that of neat PE-RT, whereas GNP1.0 showed higher crystallinity but poorer barrier performance. Thus, the permeability trend does not follow the variation of bulk crystallinity. The Nielsen analysis yielded an effective aspect ratio of approximately 570 for GOS1.0, supporting an important contribution from the platelet-induced tortuous diffusion pathways. However, this value is a model-derived transport parameter rather than a direct measurement of platelet dimensions, exfoliation, or nanoscale dispersion. The results therefore suggest that filler surface chemistry, interfacial characteristics, and platelet arrangement contribute to the observed barrier response, although their individual contributions cannot be quantitatively resolved.
(2) GNP exhibited the highest mean yield strength and elastic modulus at 1 wt %, with increases of approximately 26.4% and 50.8%, respectively, relative to neat PE-RT. GO also produced substantial increases in the yield strength (+20.3%) and elastic modulus (+36.1%), whereas GOS and rGO showed more moderate changes. These results indicate that the structural factors governing mechanical reinforcement differ from those controlling the gas-barrier performance.
(3) Six months of immersion in synthetic hydrocarbon oil produced formulation-dependent changes in thermal, dimensional, surface, and molar mass-related properties. The graphene-containing nanocomposites generally showed reduced dimensional changes compared with neat PE-RT, while GOS exhibited comparatively stable GPC parameters after 6 months. These observations indicate differences in the aging response among the investigated formulations; however, the underlying stabilization or degradation mechanisms were not directly determined. In particular, radical-scavenging activity was not experimentally evaluated and, therefore, cannot be established as the mechanism responsible for the observed behavior.
(4) When CO_2_ barrier, mechanical, and aging performance are considered together, GOS at 1 wt % provided the best overall balance among the formulations and compositions investigated. Although GNP1.0 exhibited the highest mean elastic modulus, its comparatively poor CO_2_ barrier performance limited its suitability when gas transport is a primary requirement. GOS1.0 combined the largest CO_2_ permeability reduction with an increased stiffness relative to neat PE-RT and comparatively stable behavior during hydrocarbon aging.

The present results should be interpreted as a comparative assessment under the investigated processing and aging conditions rather than as a prediction of long-term field performance. Future work should address scale-up of GOS compounding to corotating twin-screw geometries, longer-term aging under combined hydrocarbon, CO_2_, water, pressure, thermal cycling, and mechanical loading conditions representative of flexible riser service, and direct spectroscopic quantification of oxidation progression in aged samples.

## Supplementary Material



## Notes

XRD structural parameters (2θ, d_002_, and crystallite size *L*_c_) of graphite, GO, rGO, GOS, and GNP nanofillers (**Table S1**); DSC thermal parameters (*T*_c_, *T*_m_, Δ*H*_m_, and degree of crystallinity *X*_c_) of unaged PE-RT nanocomposites (**Table S2**); tensile mechanical properties (elastic modulus, elongation and stress at yield, and elongation and stress at break) of nanocomposites at 1 wt % filler loading (**Table S3**); sensitivity analysis of the Nielsen model for IGOS1.0 as a function of effective platelet aspect ratio (**Table S4**); TGA parameters (*T*_10%_, *T*_onset_, *T*_max_, and residue) of GO-, rGO-, and GOS-containing nanocomposites during 6 months of aging in synthetic oil at 80 °C (**Table S5**); comparative thermal stability of the nanocomposites before and after 6 months of aging (**Table S6**); DSC thermal parameters (*T*_m_, *T*_c_, Δ*H*_m_, and *X*_c_) of nanocomposites during aging (**Table S7**); GPC molar mass parameters (*M*_n_, *M*_w_, and dispersity Đ) of nanocomposites before and during aging (**Table S8**); evolution of TGA parameters (*T*_10%_, *T*_onset_, and *T*_max_) during aging (**Figure S1**); DSC thermograms of aged nanocomposites (**Figure S2**); evolution of GPC molar mass parameters (*M*_n_, *M*_w_, and *Đ*) during aging (**Figure S3**); and CO_2_ diffusion and permeability calculation methodology (**Equations S1–S3**) (PDF)
