# Supplementary material for: Graphene-Reinforced PE-RT Nanocomposites for Flexible Pipeline Barriers: Structure–Property–Performance and Aging
Source: ACS Omega. 2026 Oct 14;11(42):65747–64. doi: 10.1021/acsomega.6c06321 (PMC13718216; doi:10.1021/acsomega.6c06321)
Supplement: Supplementary file 1 [file ao6c06321_si_001.pdf]

SUPPORTING INFORMATION

Graphene-Reinforced PE-RT Nanocomposites for Flexible Pipeline Barriers: Structure–Property–Performance and Aging

Lucas Galhardo Pimenta Tienne,[1] Barbara de Salles Macena da Cruz,[1] Vinicius de Oliveira Aguiar,[1] Fábio Elias Jorge,[1] Maria de Fátima Vieira Marques,[1]* Erica Gervasoni Chaves[2]

[1]*Institute of Macromolecules Professor Eloisa Mano (IMA), Federal University of Rio de Janeiro, Av. Horácio Macedo 2090, Rio de Janeiro, CEP 21941-598, RJ, Brazil*

[2]*Leopoldo Americo Miguêz de Mello Research and Development Center, PETROBRAS/CENPES, Av. Horácio Macedo 950, Rio de Janeiro, CEP 21941-915, RJ, Brazil*

*Corresponding author: fmarques@ima.ufrj.br

Table S1: XRD parameters of graphene-based nanofillers.

SAMPLE	2θ (°)	d_{002} (nm)	L_C (nm)
Graphite	26.43	0.34	22.60
GO	26.35*	0.34*	6.20*
rGO	26.59	0.33	5.90
GOS	26.07*	0.34*	7.50*
GNP	26.65	0.33	19.10

*Deconvoluted plane in the region near 26°.

Table S2: DSC data for unaged nanocomposites.

SAMPLE	T_c (°C)	T_m (°C)	ΔH_m (J/g)	X_c (%)
Intrepid	116	130	145	49.4
IGO0.5	116	130	194	66.5
IGO1.0	117	130	144	49.7
IGO2.0	117	130	160	55.7
IrGO0.5	117	130	192	65.9
IrGO1.0	117	129	139	47.9
IrGO2.0	117	129	153	53.2
IGOS0.5	117	130	136	46.6
IGOS1.0	117	130	158	54.4
IGOS2.0	117	130	148	51.5
IGNP0.5	118	129	154	52.8
IGNP1.0	116	131	178	61.3
IGNP2.0	116	131	125	43.5

Table S3: Tensile mechanical properties at 1 wt%.

SAMPLE	Young Modulus or Elastic Modulus (MPa)	Elongation at yield strength εy (%)	Stress at yield strength σy (MPa)	Elongation at break εr (%)	Tension at break σr (MPa)
Intrepid	301.3 ± 31.7	13.3 ± 0.9	18.2 ± 1.3	491.7 ± 67.3	20.0 ± 2.1
IGO1.0	410.0 ± 54.6	9.8 ± 0.7	21.9 ± 1.4	336.8 ± 62.8	19.1 ± 3.0
IrGO1.0	348.4 ± 63.8	10.0 ± 0.2	19.6 ± 1.8	382.1 ± 48.9	18.4 ± 1.7
IGOS1.0	376.4 ± 12.9	10.1 ± 0.4	20.8 ± 1.0	286.0 ± 167.8	18.6 ± 4.0
IGNP1.0	454.5 ± 30.7	10.3 ± 0.4	23.0 ± 1.0	312.9 ± 134.8	19.8 ± 5.3

Table S4: Sensitivity analysis of the Nielsen model for IGOS1.0 as a function of effective platelet aspect ratio.

**Effective aspect ratio, α**	**Predicted P/P_0**	**Predicted permeability reduction (%)**
50	0.895	10.5
100	0.813	18.7
200	0.685	31.5
300	0.590	41.0
400	0.516	48.4
500	0.458	54.2
570	0.436	56.4
700	0.387	61.3
1000	0.306	69.4

Calculation based on the Nielsen equation using φ =0.0045 for IGOS1.0.

Table S5: TGA data for aged nanocomposites.

SAMPLE	$T_{10\%}$ (°C)	T_{onset} (°C)	T_{max} (°C)	Residue (%)
Intrepid	431 ± 2	446 ± 3	474 ± 2	0.08
IGO1.0	442 ± 3	456 ± 3	477 ± 5	1.02
IGO1.0 15D	428 ± 4	442 ± 5	462 ± 4	0.79
IGO1.0 1M	435 ± 5	443 ± 5	468 ± 6	1.45
IGO1.0 2M	443 ± 4	456 ± 5	479 ± 4	1.11
IGO1.0 3M	427 ± 5	438 ± 5	465 ± 6	0.97
IGO1.0 4M	437 ± 6	450 ± 6	473 ± 5	1.45
IGO1.0 5M	443 ± 5	455 ± 5	476 ± 6	1.29
IGO1.0 6M	440 ± 6	455 ± 6	477 ± 5	1.51
IrGO1.0	436 ± 4	451 ± 4	474 ± 6	0.40
IrGO1.0 15D	436 ± 3	446 ± 4	465 ± 3	0.99
IrGO1.0 1M	422 ± 5	434 ± 5	463 ± 6	1.46
IrGO1.0 2M	450 ± 7	461 ± 5	481 ± 5	1.56
IrGO1.0 3M	446 ± 5	458 ± 5	479 ± 5	0.81
IrGO1.0 4M	447 ± 4	456 ± 4	476 ± 3	0.68
IrGO1.0 5M	445 ± 5	457 ± 5	478 ± 3	0.85
IrGO1.0 6M	432 ± 4	443 ± 4	468 ± 4	0.30
IGOS1.0	441 ± 5	455 ± 5	477 ± 6	0.60
IGOS1.0 15D	435 ± 3	450 ± 3	475 ± 4	0.77
IGOS1.0 1M	435 ± 5	442 ± 5	468 ± 5	1.45
IGOS1.0 2M	450 ± 6	461 ± 5	481 ± 5	1.56
IGOS1.0 3M	427 ± 5	438 ± 5	465 ± 6	0.97
IGOS1.0 4M	447 ± 4	456 ± 4	476 ± 3	0.68
IGOS1.0 5M	443 ± 4	455 ± 3	476 ± 3	1.30
IGOS1.0 6M	432 ± 3	443 ± 2	468 ± 4	0.80

Table S6: Comparative thermal stability before and after 6 months aging.

**Sample**	**T_{onset} (°C)**			**T_{max} (°C)**			**T_{onset} at 6M vs. Intrepid unaged (°C)**
	Unaged	6 M	ΔT_{onset}[a]	Unaged	6 M	ΔT_{max}[a]	
IGO1.0	456	455	−1	477	477	0	+9
IrGO1.0	451	443	−8	474	468	−6	−3
IGOS1.0	455	443	−12	477	468	−9	−3
Reference							
Intrepid (unaged)	446	n/a[b]	—	474	n/a[b]	—	—

[a] $\Delta T = T_{6M} - T_{unaged}$; negative values indicate a decrease after aging. Measurement uncertainty: ±3–6 °C (see Table S5).

[b] Neat PE-RT was not included in the TGA aging series (Table S5); degradation of the unfilled matrix was assessed by visual inspection, mass uptake (Fig. 11), and contact angle (Fig. 14), all of which confirmed severe thermo-oxidative deterioration.

Table S7: DSC data for aged nanocomposites.

SAMPLE	T_m (°C)	T_c (°C)	ΔH_m (J/g)	X_c (%)
Intrepid	130	116	145.0	49.4
IGO1.0	130	117	144.0	49.7
IGO1.0 15D	129	117	151.9	52.3
IGO1.0 1M	129	117	173.6	59.9
IGO1.0 2M	129	117	174.1	60.0
IGO1.0 3M	128	117	140.0	48.2
IGO1.0 4M	129	117	151.8	52.3
IGO1.0 5M	129	117	153.9	53.0
IGO1.0 6M	130	116	175.3	60.4
IrGO1.0	129	117	139.0	47.9
IrGO1.0 15D	130	116	156.5	53.9
IrGO1.0 1M	129	117	120.2	41.4
IrGO1.0 2M	128	117	169.9	58.5
IrGO1.0 3M	129	116	157.7	54.3
IrGO1.0 4M	129	117	173.4	59.7
IrGO1.0 5M	129	116	149.9	51.6
IrGO1.0 6M	129	116	112.5	38.7
IGOS1.0	130	117	158.0	54.4
IGOS1.0 15D	129	117	171.9	59.2
IGOS1.0 1M	129	117	170.1	58.6
IGOS1.0 2M	129	117	122.4	42.1
IGOS1.0 3M	129	117	154.7	53.3
IGOS1.0 4M	129	117	226.4	78.0
IGOS1.0 5M	129	116	149.8	51.6
IGOS1.0 6M	129	116	133.2	45.9

Table S8: GPC data before and after aging.

SAMPLE	M_n x 10^{-3}	M_w x 10^{-3}	Đ Average
IGO1.0	32	546	17
IGO1.0 1M	20	262	13
IGO1.0 2M	38	395	10
IGO1.0 3M	34	322	9
IGO1.0 4M	34	247	7
IGO1.0 5M	33	398	12
IGO1.0 6M	29	269	9
IrGO1.0	33	221	6
IrGO1.0 1M	33	258	8
IrGO1.0 2M	36	501	14
IrGO1.0 3M	33	523	16
IrGO1.0 4M	28	333	12
IrGO1.0 5M	29	359	12
IrGO1.0 6M	31	288	9
IGOS1.0	26	493	18
IGOS1.0 1M	32	494	15
IGOS1.0 2M	28	491	18
IGOS1.0 3M	25	491	20
IGOS1.0 4M	26	474	18
IGOS1.0 5M	36	503	14
IGOS1.0 6M	25	520	21

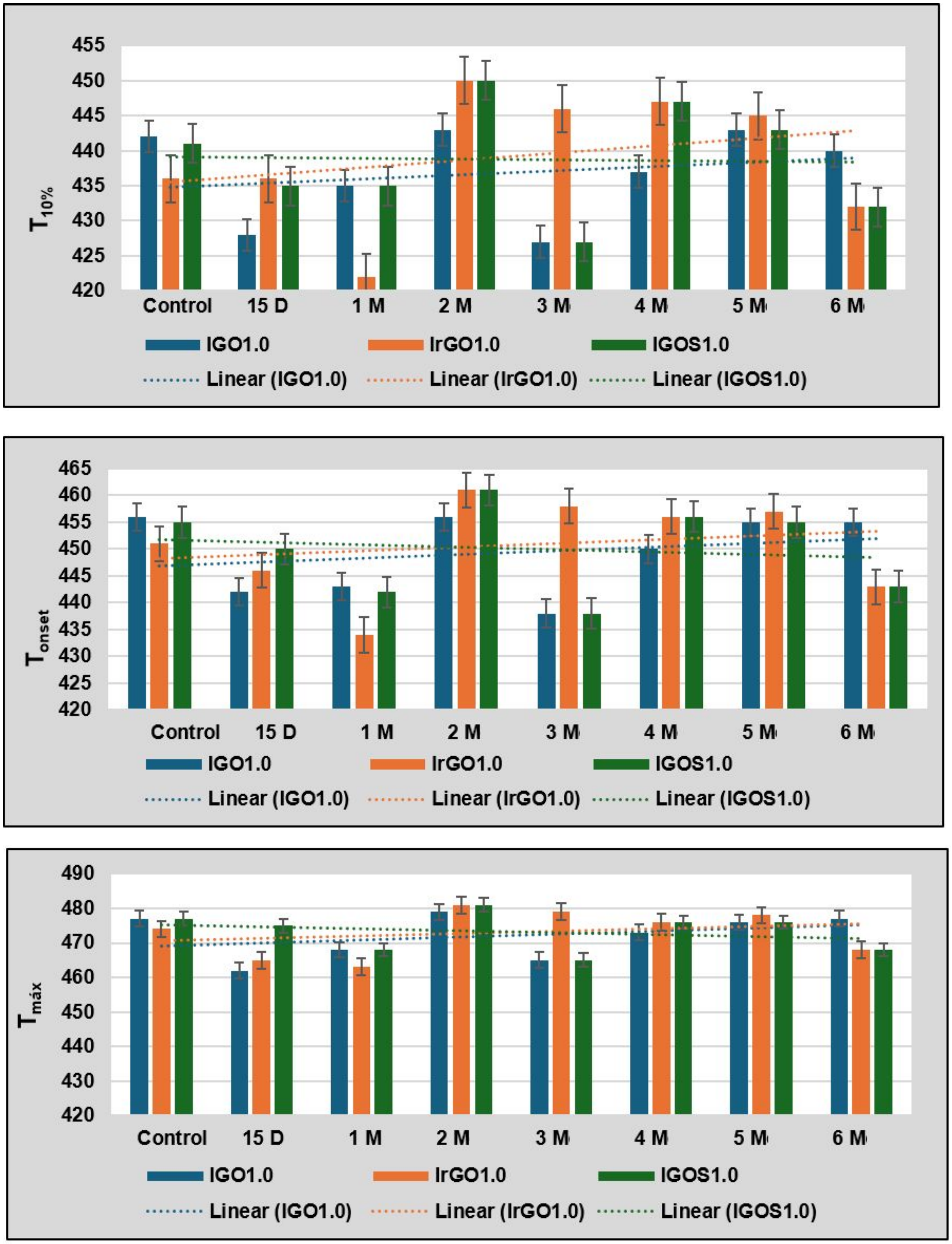

Figure S1: TGA parameters of PE-RT nanocomposites during aging in synthetic oil at 80 °C: (a) temperature at 10% mass loss ($T_{10\%}$), (b) onset degradation temperature (T_{onset}), and (c) maximum degradation-rate temperature (T_{max}). Error bars represent the experimental uncertainty.

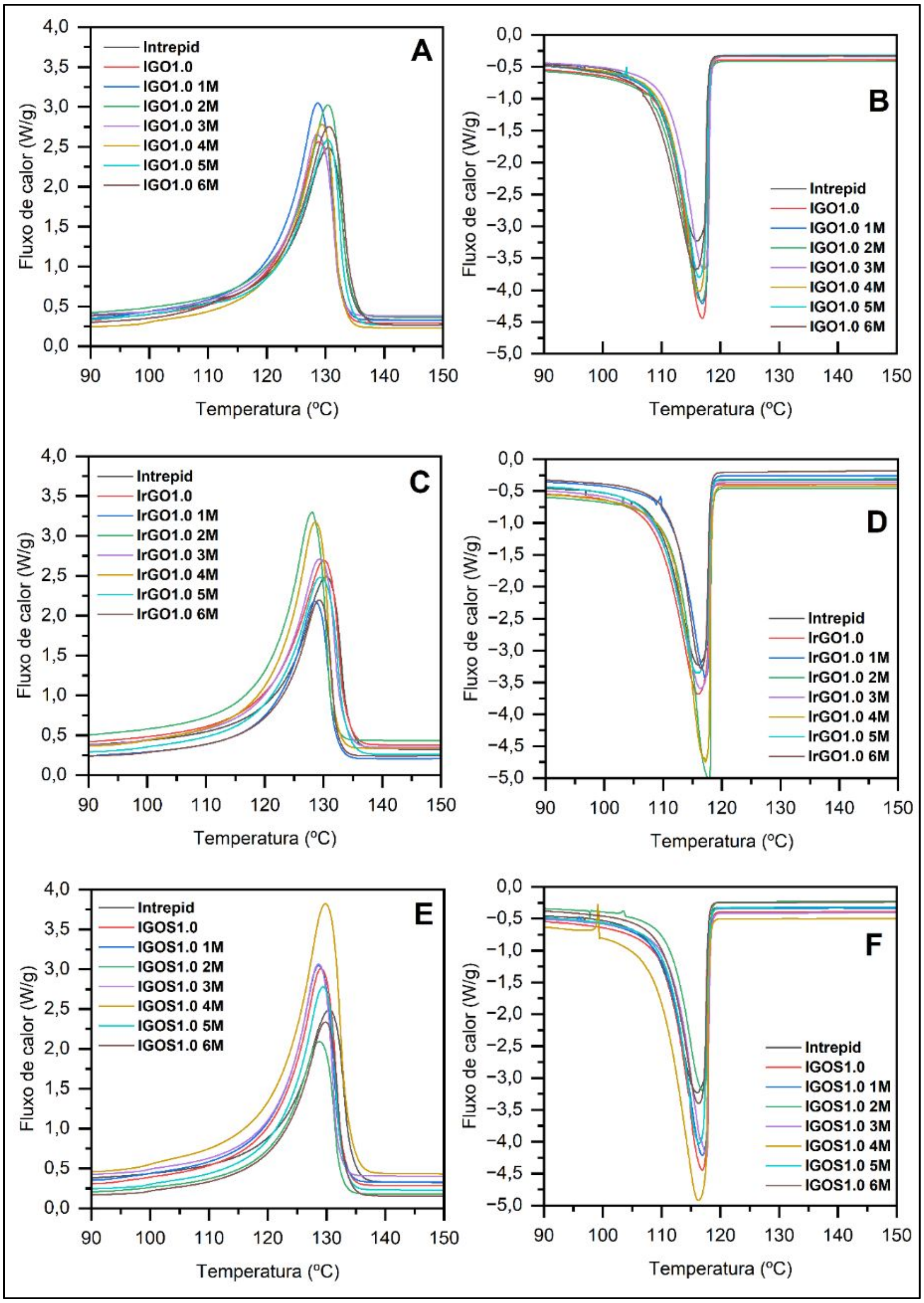

Figure S2: DSC thermograms of neat Intrepid and PE-RT/graphene-based nanocomposites before and after aging for 1–6 months: (a) IGO, heating curves; (b) IGO, cooling curves; (c) IrGO, heating curves; (d) IrGO, cooling curves; (e) IGOS, heating curves; and (f) IGOS, cooling curves.

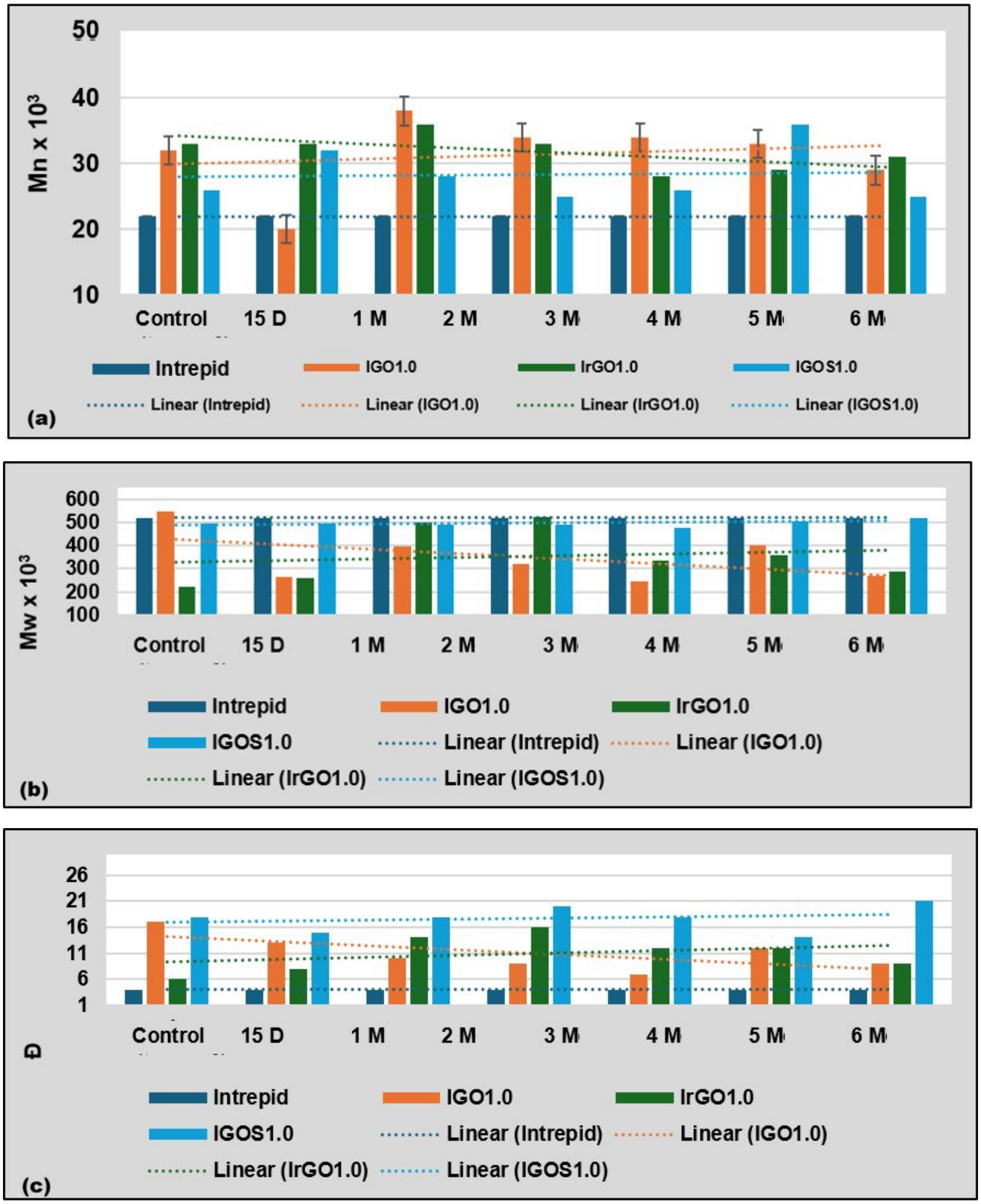

Figure S3: Evolution of the molar-mass distribution of PE-RT nanocomposites during aging in synthetic oil at 80 °C: (a) number-average molar mass (Mₙ), (b) weight-average molar mass (M_v), and (c) dispersity (Đ).

Equations S1–S3: CO_2 permeability calculation methodology.

Diffusion coefficient (Di) was calculated using Equation 5, considering the sample thickness (l) and the pressure stabilization time (θ) between the two sides of the cell after gas application.

$$D_i = l^2 / 6\theta \ (cm^2/s) \qquad \text{Equation S1}$$

Permeability coefficient (K) was obtained from Equation 6,

$$K = l \times (dP/dt) \times (V_{Perm}/(A_{Perm} \times P_i)) \times T_{CNTP}/ (T_{Exp} \times P_{CNTP}) \qquad \text{Equation S2}$$

where (V_{perm}) was calibrated by bubble-meter measurements and correlated with the pressure-increase rate (dP/dt). Ten calibration runs (100 bubble counts) established the volumetric flow rate. Standard-condition parameters (PCNTP and TCNTP) were applied for normalization. The calibrated permeation volume was obtained using Equation 7.

$$V_{perm} = (dV/dt) / (dP/dt) \qquad \text{Equation S3}$$
